# Trophoblast-derived miR-410-5p induces M2 macrophage polarization and mediates immunotolerance at the fetal-maternal interface by targeting the STAT1 signaling pathway

**DOI:** 10.1186/s12967-023-04831-y

**Published:** 2024-01-04

**Authors:** Jing Yang, Longfei Li, Linlin Wang, Ruizhi Chen, Xiaobing Yang, Juanhua Wu, Gang Feng, Jinli Ding, Lianghui Diao, Jiao Chen, Jing Yang

**Affiliations:** 1 Reproductive Medical Center, Renmin Hospital of Wuhan University & Hubei Clinic Research Center for Assisted Reproductive Technology and Embryonic Development, Wuhan, 430060 Hubei People’s Republic of China; 2 Department of Gynecology, Affiliated Cancer Hospital of Xinjiang Medical University, Urumqi, 830000 Xinjiang People’s Republic of China; 3 Shenzhen Key Laboratory of Reproductive Immunology for Peri-Implantation, Shenzhen Zhongshan Institute for Reproductive Medicine and Genetics, Guangdong Engineering Technology Research Center of Reproductive Immunology for Peri-Implantation, Shenzhen Zhongshan Obstetrics & Gynecology Hospital (Formerly Shenzhen Zhongshan Urology Hospital), Shenzhen, 518045 Guangdong People’s Republic of China; 4 Department of Clinical Laboratory, Shenzhen Zhongshan Obstetrics & Gynecology Hospital (Formerly Shenzhen Zhongshan Urology Hospital), Shenzhen, 518045 Guangdong People’s Republic of China; 5 Department of Gynecology, Shenzhen Zhongshan Obstetrics & Gynecology Hospital (Formerly Shenzhen Zhongshan Urology Hospital), Shenzhen, 518045 Guangdong People’s Republic of China

**Keywords:** Spontaneous miscarriage, Fetal-maternal cross-talk, miR-410-5p, Macrophage polarization, Immunotolerance

## Abstract

**Background:**

Macrophages phenotypic deviation and immune imbalance play vital roles in pregnancy-associated diseases such as spontaneous miscarriage. Trophoblasts regulate phenotypic changes in macrophages, however, their underlying mechanism during pregnancy remains unclear. Therefore, this study aimed to elucidate the potential function of trophoblast-derived miRNAs (miR-410-5p) in macrophage polarization during pregnancy.

**Methods:**

Patient decidual macrophage tissue samples in spontaneous abortion group and normal pregnancy group (those who had induced abortion for non-medical reasons) were collected at the Reproductive Medicine Center of Renmin Hospital of Wuhan University from April to December 2021. Furthermore, placental villi and decidua tissue samples were collected from patients who had experienced a spontaneous miscarriage and normal pregnant women for validation and subsequent experiments at the Shenzhen Zhongshan Obstetrics & Gynecology Hospital (formerly Shenzhen Zhongshan Urology Hospital), from March 2021 to September 2022. As an animal model, 36 female mice were randomly divided into six groups as follows: naive-control, lipopolysaccharide-model, agomir-negative control prevention, agomir-410-5p prevention, agomir-negative control treatment, and agomir-410-5p treatment groups. We analyzed the miR-410-5p expression in abortion tissue and plasma samples; and supplemented miR-410-5p to evaluate embryonic absorption in vivo. The main source of miR-410-5p at the maternal–fetal interface was analyzed, and the possible target gene, signal transducer and activator of transcription (STAT) 1, of miR-410-5p was predicted. The effect of miR-410-5p and STAT1 regulation on macrophage phenotype, oxidative metabolism, and mitochondrial membrane potential was analyzed in vitro.

**Results:**

MiR-410-5p levels were lower in the spontaneous abortion group compared with the normal pregnancy group, and plasma miR-410-5p levels could predict pregnancy and spontaneous abortion. Prophylactic supplementation of miR-410-5p in pregnant mice reduced lipopolysaccharide-mediated embryonic absorption and downregulated the decidual macrophage pro-inflammatory phenotype. MiR-410-5p were mainly distributed in villi, and trophoblasts secreted exosomes-miR-410-5p at the maternal–fetal interface. After macrophages captured exosomes, the cells shifted to the tolerance phenotype. STAT1 was a potential target gene of miR-410-5p. MiR-410-5p bound to STAT1 mRNA, and inhibited the expression of STAT1 protein. STAT1 can drive macrophages to mature to a pro-inflammatory phenotype. MiR-410-5p competitive silencing of STAT1 can avoid macrophage immune disorders.

**Conclusion:**

MiR-410-5p promotes M2 macrophage polarization by inhibiting STAT1, thus ensuring a healthy pregnancy. These findings are of great significance for diagnosing and preventing spontaneous miscarriage, providing a new perspective for further research in this field.

**Supplementary Information:**

The online version contains supplementary material available at 10.1186/s12967-023-04831-y.

## Introduction

The maternal–fetal interface is a complex system involving various networks, including immunological, metabolic, and neuroendocrine networks. Trophoblasts are fetal-originated cells that are crucial in establishing and maintaining pregnancy [1, 2]. The ability of a mother’s immune system to tolerate the semi-allogeneic fetal graft is the foundation of a successful pregnancy, achieved by the synergistic action of multiple cells, proteins, and nucleic acids at the interface [3]. However, the molecular mechanisms underlying fetal tolerance remain poorly understood. Throughout pregnancy, several modulatory massagers, including costimulatory molecules T cell immunoglobulin mucin 3 (Tim-3), galactose lectin-9 (Gal-9), histocompatibility leukocyte antigen (HLA)-G, interleukin (IL)-34, IL-35, chemokine ligand 16 (CXCL16), CCL2, programmed death ligand 1 (PD-L1), and programmed death-1 (PD1) have been reported to regulate the immunological functions of human macrophages, natural killer (NK) cells, dendritic cells (DC), or T cells, maintaining maternal and infant immune tolerance [4–7]. In addition, IL-33, IL-35, and toll-like receptor (TLR) 4 are also key signaling molecules that regulate uterine tissue remodeling and immune cell function in early pregnancy in mice [4, 8, 9]. Notably, trophoblasts play a vital role in the development of the placenta and the consequent formation of nutritional interactions at the maternal–fetal interface through direct or indirect ways involving the release of placental miRNAs. The unique miRNAs released by trophoblast cells are packed in extracellular vesicles (EVs) or bound to protein complexes, functioning as regulatory signals between the fetal graft and maternal immune systems. These miRNAs can regulate the adhesion and migration ability of maternal epithelial and stromal cells, change the function of the endometrium, stimulate T and B cells activation through antigen receptors, or affect the amplification and differentiation of Tregs [10, 11].

Previous studies have confirmed that the T cell type 2 (Th2) bias environment at the maternal–fetal interface participates in the maintenance of a successful pregnancy; however, the Th1 bias environment is associated with pregnancy loss [12]. After pregnancy arises, monocytes are recruited to the maternal–fetal interface, where they differentiate and mature into decidual macrophages, which are involved in regulating immune tolerance at the interface and promoting placental formation [13]. Macrophages account for approximately 20% of all decidual leukocytes in the first trimester of pregnancy [4]. Interestingly, decidual macrophages are primarily present in the M2 immunophenotype, which inhibits allogeneic fetal antigen response by producing immunosuppressive cytokines and phagocytosis of apoptotic trophoblast cells, among others [4, 6]. Decidual macrophages not only prevent the release of pro-inflammatory substances and inhibit the cytotoxic function of NK cells but also convert helper Th1 immune responses into Th2-type immune responses, which helps create a tolerant immune environment [6, 12]. In early pregnancy, a dominant polarization towards M2 macrophages is necessary for a successful pregnancy, while the imbalance of M1/M2 during pregnancy is associated with spontaneous abortion [14–16].

EVs and exosomes (EXOs) play crucial roles in intercellular communication. EXOs are a group of nanoparticles (50–200 nm in diameter) formed by endosomal membrane germination. The double-layer structure of the extracellular membrane protects the inner molecules from degradation in the extracellular environment. Recently, EXOs derived from fetal grafts have been reported to play a principal role in maintaining maternal–fetal immune tolerance [17]. Increasing evidence has demonstrated that EXOs can transfer numerous proteins and various nucleic acids (mRNAs, miRNAs, LncRNAs, and CircRNAs) to recipient cells and consequently regulate their biological activities [18–20]. Among the numerous cargos, miRNAs are endogenous short (21–24 nucleotides long) non-coding RNA, regulating the expression of target genes by binding to the 3-untranslated region (3ʹ-UTR) of mRNAs for degradation or inhibition of translation [21]. Notably, the abnormal expression of miRNAs is related to pregnancy complications such as spontaneous abortion [20, 22], which may be involved in decidual macrophage polarization [23, 24].

Trophoblasts underlying mechanism during pregnancy remains unclear. However, given the important role of EXOs in intercellular interaction between macrophages and trophoblasts at the maternal–fetal interface, we speculated that fetal trophoblasts regulate the phenotype and function of decidual macrophages by secreting EXOs-miRNA, consequently promoting the establishment of maternal immune tolerance and tissue homeostasis. Therefore, this study aimed to elucidate the potential function of trophoblast-derived miRNAs in macrophage polarization during pregnancy.

## Methods

### Patients sample collection and preparation

The decidual macrophage samples were collected from 13 patients who had experienced unexplained recurrent spontaneous abortion (RSA) and 13 who had experienced a normal pregnancy (NP) and had induced abortion for non-medical reasons. All samples were from 6 to 10 weeks' gestation and were collected at the Reproductive Medicine Center of Renmin Hospital of Wuhan University from April to December 2021. Three cases in each group were analyzed via the small regulatory RNA (sRNA) sequencing, commissioned by RiboBio (Guangzhou, China), and the remaining 10 cases were validated using miRNA quantitative real-time polymerase chain reaction (qRT-PCR). The experiments were approved by the Renmin Hospital Research and Ethics Committee (Approval number: WDRY2020-K218), and all patients provided informed consent.

Furthermore, placental villi and decidua tissue samples were collected for validation and subsequent experiments at the Shenzhen Zhongshan Obstetrics and Gynecology Hospital (formerly Shenzhen Zhongshan Urology Hospital), from March 2021 to September 2022. Samples of placental villi and decidua tissues from 21 patients with spontaneous miscarriage (SM) and 20 NP women who had induced abortion for non-medical reasons were collected during the operation. Patients with endocrine or metabolic diseases, uterine abnormalities, infections, and fetal chromosome abnormalities were excluded from the study. All samples were approved by Research Ethics Committee of Shenzhen Zhongshan Obstetrics and Gynecology Hospital (formerly Shenzhen Zhongshan Urology Hospital) (Approval Number: SZZSECHU-2020011), and informed consent was obtained from all patients.

The villi were cut into three parts. One part was made into formalin-fixed paraffin-embedded (FFPE) tissues, the second part was frozen at – 80 °C, and the last was isolated for placental explant culture. The collected decidual tissues were cut into two parts: one part was made into FFPE and the other was frozen in liquid nitrogen. Plasma from 17 non-pregnant women (who had given birth normally), 31 early pregnant women (who had 6–10 weeks gestation), 30 women with threatened abortion (TA), and 30 women who had SM was collected by centrifugation (3500 rpm/min, 10 min). Table 1 summarizes the baseline characteristics of the patients.

**Table 1:** Clinical characteristics of subjects

Parameter		Number	Age (years, mean ± SEM)	Gestation week (mean ± SEM)
Operation group	NP	19	32.61 ± 1.70	7.20 ± 0.21
	SM	21	33.95 ± 1.15	7.15 ± 0.24
Plasma group	NoP	17	33.24 ± 1.19	–
	EP	30	32.77 ± 0.47	7.28 ± 0.37
	TA	30	32.20 ± 0.86	6.85 ± 0.21
	SM	30	33.57 ± 0.64	7.31 ± 0.13

*NP* normal pregnant, *SM* Spontaneous miscarriages, *NoP* No pregnancy, *EP* Early pregnancy, *TA* Threatened abortion

### Placental explant culture

Approximately 200 mg of fresh villous tissues were washed with aseptic phosphate-buffered saline (PBS) and cultured in an EXOs-removed cell DMEM-F12 medium containing 10% fetal bovine serum (FBS) and 1% Penicillin–Streptomycin-Amphotericin B. Subsequently, the explants were cultured at 37 ℃ and 2% oxygen to simulate the in vivo environment of placental development. After 3 h, the culture medium was replaced to remove cell and apoptotic fragments, and the cells were cultured for another 40 h before collecting the EXOs. Unless otherwise noted, information on the chemicals and reagents used in this study is provided in Additional file 1: Table S1.

### MiRNA Fluorescence in situ hybridization (FISH)

The miRNA FISH assay was performed based on the instructions provided by BersinBio^™^ for the FISH Kit. Paraffin sections were incubated and closed at 37 °C for 30 min after dewaxing and dehydration. Then the sections were co-altered at 73 °C for 5–8 min after dropwise addition of hybridization reaction solution and quickly transferred to 37 °C for hybridization overnight. After adding DAPI and the anti-fluorescence attenuation mounting agent, the sections were placed under a Leica TCS SP8 Laser Confocal Scanning Microscope (Leica Biosystems, Wetzlar, Germany) for observation.

### Cell culture

Human trophoblast cell lines HTR8/SVneo, JEG3, and monocyte THP1, as well as mouse macrophage RAW264.7, were purchased from Wuhan Procell Life Science and Technology Co., Ltd., China. The human trophoblast cell line, termed JAR, and human embryonic kidney cell line, entitled 293 T, were purchased from the Cell Bank of the Chinese Academy of Sciences. HTR8/SVneo cells were cultured in a DMEM-F12 medium. JAR and THP1 cells were cultured in RPMI-1640 medium. JEG3 cells were cultured in a MEM medium. 293 T and RAW264·7 cells were grown in DMEM medium. All culture media contained 10% FBS and 1% penicillin–streptomycin. For the THP1 cell, 0·05 mM β-mercaptoethanol was added to the medium. Unless otherwise stated, all cells used throughout the experiments were maintained in an incubator containing 5% CO_2_ at 37 ℃.

THP1 monocytes were matured into M0 macrophages with 50 ng/mL of phospholipid 12-myristic acid 13-acetate (PMA) for 24 h. M0 macrophages were polarized into M1 macrophages with 100 ng/mL of lipopolysaccharide (LPS) and 20 ng/mL of interferon γ (IFN-γ) or M2 macrophages by stimulating 20 ng/mL of IL-4 and 20 ng/mL of IL-13 for 48 h. For macrophage and trophoblast cell co-culture experiments, trophoblast cells were inoculated in the upper side of a 0·4 μm insert (Corning, NY, USA), while macrophages were seeded in the bottom of 6-wells or 12-wells plate.

### EXOs extraction

EXOs from the placental villi or trophoblast cells were collected using the TransExo™ Cell Media Exosome Kit. Briefly, the culture medium was centrifuged at 3000 g for 30 min at 4 ℃ to remove dead cells and cell fragments. The supernatant was filtered through a 0·22 μm membrane to remove large granular vesicles. After mixing with the extraction reagent overnight at 4 ℃, the EXOs were collected by centrifugation (10,000 g, 30 min) at 4 ℃. The obtained EXOs resuspended in PBS were characterized or stored at −80 °C for the following steps.

### Characterization of EXOs

The morphology of the EXOs was observed using transmission electron microscopy. Briefly, the EXOs were fixed in 4% paraformaldehyde (PFA) and placed on a 200 mesh Formvar-carbon sample-carrying copper net. The grid was stained with 4% uranyl acetate and 2% methylcellulose and air-dried before use. Images were obtained using an HT7700 transmission electron microscope (Nippon Electron Co. Ltd., Tokyo, Japan). For the concentration and size distribution analysis, the EXOs were suspended as a 0·5% solution in 1 mL of PBS and analyzed using a Malvern Panalytical Nanosight NS300 (Spectris, London, UK). For the identification of protein markers, EXOs protein (2 μg/μL) was subjected to an automated Wes Capillary System (12–230 kDa kit) and examined by SM-W004 (Protein Simple, San Jose, CA, USA). Detailed antibody information is shown in Additional file 1: Table S2.

### Cellular internalization of EXOs

EXOs were labeled with PKH26, whereas macrophages were labelled with PKH67, according to the manufacturer’s instructions. The PKH26-labelled EXOs at a concentration of 30 μg/mL were added into the culture medium of PKH67-labelled macrophages and photographed continuously every hour for 12 h using the Operetta CLS High connotative Analysis system (PerkinElmer, MA, USA).

### EXOs blockade and cell treatment

In order to clarify the relationship between the expression level of miR-410-5p and EXOs production, GW4869, a neutral sphingomyelinase inhibitor which can prevent the formation of intraluminal vesicles, was used to block EXO production from trophoblasts. Briefly, trophoblasts were treated with 10 μM or 20 μM GW4869 for 24 h. EXOs were collected as previously described in the EXOs extraction section.

To investigate the effect of trophoblast-derived EXOs on macrophage phenotype, macrophages were seeded in 6-well or 12-well plates and treated with 30 μg/mL EXOs for 48 h. The PBS-treated group was used as the vehicle control in all cell experiments.

### Cell transfection

In all cell transfections, oligonucleotides, including miR-410-5p mimic, mimic-NC, miR-410-5p inhibitor, inhibitor-NC, pmirGLOSTAT1-WT, pmirGLO-STAT1-MUT, si-STAT1, or overexpressed (oe)-STAT1, were introduced into cells through liposome 2000 with a final oligonucleotide concentration of 20 nM, according to the manufacturer’s instructions. Additional file 1: Table S3 specifies the various sequences of oligonucleotides used in this study.

### RNA isolation and quantitative real-time polymerase chain reaction (qRT-PCR)

Plasma miRNAs were extracted using the miRcute serum plasma miRNA extraction and separation kit. Briefly, 0·5 μg of total RNA was reverse transcribed with the miRNA 1st Strand cDNA Synthesis Kit. Subsequent quantification was performed using miRNA Universal SYBR qPCR Master Mix, and the relative expression levels of miRNA were normalized to that of the internal control U6.

The total RNAs in the tissues and cells were isolated using TRIzol Reagent, according to the manufacturer's instructions. Briefly, 0·5 μg of RNA was reverse transcribed with the RT reagent kit, and the cDNA was used for qRT-PCR using the SYBR-Green PCR Mix by QuantStudio 5 Real-Time PCR system (Applied Biosystems, MA, USA). The relative expression of the genes was normalized to the level of β-actin expression in each sample.

The amplification reaction was performed in triplicate for all the experiments. Relative RNA quantification was performed using the comparative 2 − ΔΔCt method. Additional file 1: Table S4 details the primer sequences.

### Simple western

Cells were harvested and lysed using radioimmunoprecipitation (RIPA) lysis buffer, and the supernatant was collected from the lysates that were centrifuged at 4 °C 12000 g for 15 min. A bicinchoninic acid (BCA) assay kit was used to quantify the protein concentrations, and the automated Wes Capillary System was used to detect protein levels. Total protein loading was 0·75 μg/μL. The ratio of the gray value of the target protein to the gray value of the internal reference protein measured by the system is the relative expression amount of the protein (relative units). Detailed information regarding the antibodies used is included in Additional file 1: Table S2.

### Immunocytochemistry

The cells were incubated with mouse anti-CD68, rabbit anti-CD80, and rabbit anti-CD163 antibodies at 4 °C overnight after being fixed with 4% PFA for 30 min and blocked with 10% of bovine serum albumin for another 30 min. After removing unbound primary antibodies, goat anti-mouse immunoglobulin G (IgG) labelled with Alexa Fluor 488 or donkey anti-rabbit IgG labelled with Alexa Fluor 555 was added for 1 h. The nuclei were stained with an anti-fluorescence quenching 4ʹ,6-diamidino-2-phenylindol (DAPI) solution. Additional file 1: Table S5 provides information on the antibodies used. Mean fluorescence intensity (IntDen/Area) was quantitatively analyzed using Image J software.

### Determination of cytokines and chemokines

Cytokines and chemokines in the medium were assessed using a Human Cytokine G5 Antibody array following the manufacturer’s instructions. After the original data were normalized using software, fold change (differential expression multiple) was used to screen differential proteins. Gene Ontology (GO) and Kyoto Encyclopedia of Genes and Genomes (KEGG) pathway enrichment analyses were used to enrich differential proteins.

Cytokines in the cell culture supernatant were analyzed using the Multi-Analyte Flow Assay Kit of Macrophage/microglia. All samples were collected and analyzed using a flow cytometer, Beckman Coulter CytoFLEX (Beckman, IN, USA). A standard curve was constructed using the mean fluorescence intensity (MFI) values of known cytokine concentrations given in the cytokine kit. Additional file 1: Table S6 shows the sensitivity (minimum detectable concentration) and maximum detection limit of each cytokine in the cell culture medium. BioLegend’s LEGENDplex multi-factor dedicated software was used for data analysis, and the raw data of each factor expression obtained were standardized by Origin software and clustered in a heat map.

### Enzyme-linked immunosorbent assay (ELISA)

The IL-6 and tumor necrosis factor α (TNF-α) concentrations were determined using ELISA according to the kit's protocol. The absorption at 450 nm was determined using a GEN5^™^ Microplate data acquisition and analysis system (BioTek, WA, USA).

### Cell mRNA sequencing and analysis

The total RNA of the cells was extracted and inverted to synthesize complementary DNA (cDNA). After enrichment, the library was sequenced using the Illumina Novaseq 6000 (Illumina, CA, USA) platform. The differentially expressed genes (DEGs) among the samples were analyzed based on the quantitative gene expression results. Significantly enriched functional information was obtained through functional annotation databases, including GO and KEGG.

### Dual-luciferase reporter assay

The complementary binding sites between signal transducer and activator of transcription (STAT) 1 mRNA 3ʹ-UTR and miR-410-5p were predicted using TargetScan (http://www.targetscan.org/vert_71/). Wild-type (WT) and mutant (MUT) human STAT1 mRNA 3'-UTR luciferase reporter vectors were constructed into a pGL3-Luc vector (Qingke, Beijing, China). Subsequently, 293 T cells were co-transfected with 100 nM WT or MUT luciferase reporter plasmid supplemented with 100 nM miR-410-5p mimic or negative control (NC). After 24 h, the cells were collected and subjected to the Dual-Luciferase^®^ Reporter Assay System using a GEN5^™^ Microplate Data Acquisition and Analysis Systems (BioTek, WA, USA). The ratios of Firefly and Renilla luminescence were calculated and compared.

### Pull-down assay of target mRNAs of miR-410-5p

A commercial BersinBio™ miRNA pull-down kit was used to identify the target mRNAs of miR-410-5p. Briefly, macrophages (approximately 1 × 10^7^ cells) were transfected with a 100 nM biotin-labelled miR-410-5p probe (experimental group) or NC probe (NC group) for 48 h. The cells were collected using trypsin digestion, lysed with lysis buffer, hybridised, and incubated with streptavidin magnetic beads at 4 ℃ for 4 h. The isolated RNAs were examined using PCR and qPCR after elution and precipitation at − 80 ℃ overnight. The probe sequences are included in Additional file 1: Table S3**.**

### Determination of cell oxygen consumption rate (OCR) and extracellular acidification rate (ECAR)

OCR, an indicator of oxidative phosphorylation, was measured using an extracellular oxygen consumption assay, according to the manufacturer's recommendation. After specific treatments, oxygen consumption reagents were added to each well, except for the blank control. After sealing the plate with high-sensitivity mineral oil, the plate was subjected to a fluorescent plate reader (BioTek, WA, USA) at 37 ℃. The extracellular O_2_ consumption signals under excitation/emission (Ex/Em) = 380/650 nm were measured every 90 s and lasted for 120 min. We plotted the assay signal intensity versus time (min) and apply linear regression to determine the slope and correlation coefficient for each well. The slope represents the OCR value.

ECAR, the consequence of lactic acid production, which is a marker of glycolysis, was determined using glycolysis assay. Before the analysis, the cells were cultured without CO_2_ for 3 h to remove residual CO_2_. After adding the determination reagent, the excitation and emission wavelengths of Ex 380 nm and Em 615 nm were recorded in a fluorescent plate reader (BioTek, WA, USA) at 37 ℃. The glycolysis signal was measured every 90 s for a total of 120 min. The intensity of each well glycolysis assay versus time (min) was plotted, and linear regression was applied to determine the slope and correlation coefficient for each well. The slope obtained in this case is the ECAR value of the corresponding sample.

### Reactive oxygen species (ROS) and mitochondrial membrane potential (MMP) detection

ROS was measured using an ROS Assay Kit, according to the manufacturer's recommendations. Briefly, cells were loaded in fluorescent probe 2ʹ—7ʹ-dichlorofluorescein diacetate (DCFH-DA) and incubated in a 37 °C cell culture incubator for 20 min. After washing off the excess probes, DCF fluorescence intensity was detected under the fluorescein Isothiocyanate (FITC) channel using flow cytometry (Beckman, IN, USA). ROS in the cell can oxidize non-fluorescent DCFH to produce fluorescent DCF. Detection of the fluorescence of DCF can tell the level of ROS in the cell.

MMP was detected using the MMP Assay Kit with JC-1. The essential assay mechanism can be explained as follows. When the MMP is high, JC-1 aggregates in the matrix of mitochondria to form polymers, which can produce red fluorescence. Whereas, when the MMP is low, JC-1 cannot accumulate in the mitochondrial matrix, and JC-1 is a monomer and produce green fluorescence. Cells were incubated with JC-1 staining working solution at 37 °C for 20 min, and MMP was detected using flow cytometry (Beckman, IN, USA). The JC-1 monomer was measured using the FITC channel, while the JC-1 polymer was detected under the PE channel. The decrease MMP can be easily detected by the transition of JC-1 from red to green fluorescence, and the proportion of mitochondrial depolarization is measured by the relative ratio of red to green fluorescence.

### Mouse miscarriage model and treatments

All animal operations were approved by the ethics committee for laboratory animal welfare (IACUC) of Renmin Hospital of Wuhan University (Approval number: No. WDRM animal (f) No. 20201207). Eight-week-old female C57BL/6 mice and male BALB/c mice of the same age were provided by the Guangdong Animal Experimental Center and raised in SPF animal facilities. Overall, 36 female mice were randomly divided into six groups as follows: naive-control, LPS-model, agomir-NC prevention, agomir-410-5p prevention, agomir-NC treatment, and agomir-410-5p treatment groups. Two female C57BL/6 mice were mated with one male BALB/c mouse. The day on which the vaginal plug appeared in C57BL/6 mice was recorded as the 0·5 day (GD0·5) of pregnancy. On GD7·5, 200 μL of LPS aqueous solution (5 μg/mL) was intraperitoneally injected to induce miscarriage. For the prevention groups, three shots of 100 μL of agomir-NC or agomir-410-5p (100 mM in saline) were injected intravenously on GD0·5, GD3·5, and GD6·5. For treatment groups, three shots of 100 μL of agomir-NC or agomir-410-5p (100 mM in saline) were injected intravenously on GD8·5, GD9·5, and GD10·5. The naive-control group received an intraperitoneal injection of saline (200 μL per 20 g body weight). On GD13·5, the mice were euthanized via CO_2_ inhalation. Peripheral blood, organs (heart, liver, lung, kidney, and intestine), and placental tissues, including decidual tissues and the fetus, were collected for the following experiments.

### Hematoxylin–eosin staining

Mouse FFPE organs were used for subsequent experiments. Hematoxylin–eosin staining of FFPE sections was performed on a DRS-Prisma-P-JCS&Film-JC2 system using preset procedures (SAKURA, Osaka, Japan). The image acquisition was completed using the multi-spectral panoramic organization program analysis system on the Olympus VS200 Slide Scanner (Olympus, Tokyo, Japan).

### Immunohistochemical (IHC) staining

IHC staining was performed on FFPE sections using a Bond Polymer Refine Detection Kit (Leica Microsystems, Wetzlar, Germany) in an automatic immunostaining machine (Leica Bond Rx system, Wetzlar, Germany). The primary antibodies used are detailed in Additional file 1: Table S7, including monoclonal antibodies against CD86, CD206, and STAT1, as well as appropriate controls. Image acquisition was completed using the multi-spectral panoramic organization program analysis system on the Olympus VS200 Slide Scanner (Olympus, Tokyo, Japan) and the proportion of positive cells was analyzed using the HALO image analysis platform (Indica Labs, NM, USA).

### Statistics analysis

Unless otherwise stated, all in vitro experiments were independently performed at least thrice. Values are presented as mean ± SD for in vitro studies or mean ± SEM for clinical samples and in vivo studies. The two-tailed Student’s *t*-test and nonparametric Mann–Whitney U test were used to compare the data between any two groups based on normality and equal variance tests. One-way analysis of variance (ANOVA) with the Bonferroni post-hoc test was used for multiple comparisons. The diagnostic value was evaluated using a receiver operating characteristic (ROC) curve. Statistical analyses were performed using SPSS statistical software (version 22·0, IBM SPSS, NY, USA). Data were plotted using GraphPad Prism 6·0 software (GraphPad Software, Inc., CA, USA). In all comparisons, P values less than 0·05 were considered statistically significant.

## Results

### miR-410-5p might be a key messenger between fetal-maternal cross-talk

**Fig. 1:**
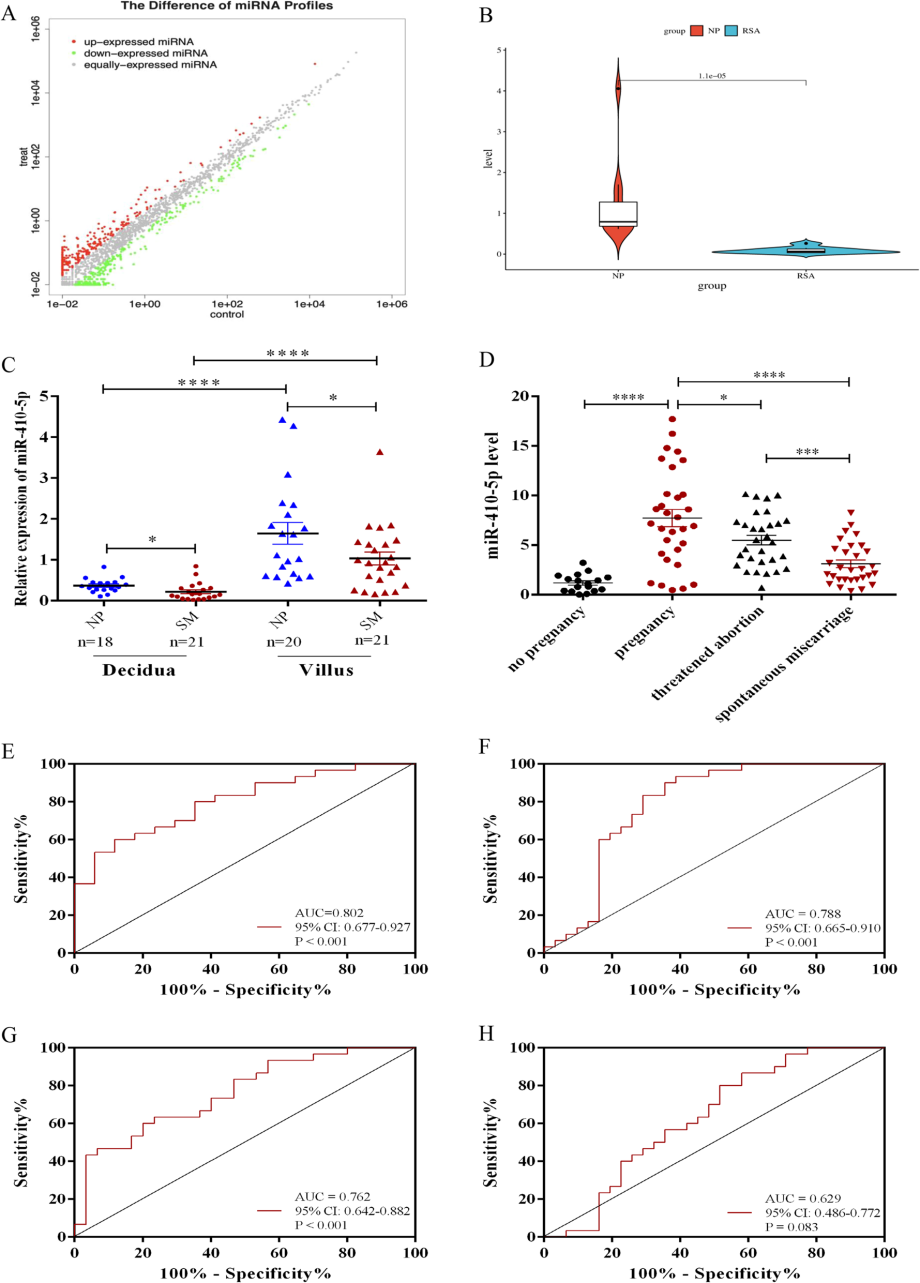
Clinical value of mir-410-5p in early pregnancy. **A** Volcano plot of differential expressed miRNA (|log2 (foldchange)|> 1 and adjusted p < 0.05). **B** Partial differential miRNA expression in decidual macrophages of induced abortion and recurrent abortion was verified by qRT-PCR. **C** The expression of miR-410-5p in decidua and villus of normal pregnancy and spontaneous abortion was detected using qRT-PCR. **D–H** The expression level of miR-410-5p in plasma and its diagnostic value. **D** qRT-PCR was used to analyze the expression of miR-410-5p in plasma of non-pregnant women (n = 17), early pregnant women (n = 31), threatened abortion group (n = 29) and spontaneous abortion group (n = 30). **E** ROC analysis of the diagnostic value of miR-410-5p in early pregnancy (early pregnancy vs non-pregnancy). **F** ROC analysis of the diagnostic value of miR-410-5p in spontaneous abortion (spontaneous abortion vs early pregnancy). **G** ROC analyzed the predictive value of miR-410-5p on spontaneous abortion (spontaneous abortion vs threatened abortion). **H** ROC analyzed the predictive value of miR-410-5p on threatened abortion (threatened abortion vs early pregnancy). Values were listed as the mean ± SEM. *P < 0·05, ***P < 0·001, ****P < 0·0001

Identifying and characterizing contributors and biomarkers for early pregnancy are important for the early diagnosis and prevention of pregnancy complications, including SM. Initially, miRNA profiles in decidual macrophages were examined via sRNA sequencing. In total, 83 miRNAs were identified as differentially expressed between the RSA and NP groups under |log2 (fold change)| larger than or equal to 1.0 and P value less than 0.05, including 47 up-expressed miRNAs and 36 down-expressed miRNAs (Fig. 1A). Fifteen different miRNAs with potent immunomodulatory actions were verified by qPCR in the other 20 decidual tissues (data was shown in Table 2). Among them, the expression of miR-410-5p exhibited similar trends with sRNA sequencing. Namely, the levels of miR-410-5p in decidual tissues from the RSA patients was significantly lower than those from the NP group (Fig. 1B). Subsequently, we observed that lower levels of miR-410-5p were present in either the decidua or villus samples from the SM group compared with the normal pregnancy group (Fig. 1C). These data suggest that miR-410-5p might be a key messenger in the fetal-maternal interface.

**Table 2:** Level of fifteen different miRNAs with potent immunomodulatory actions

	NP (n = 10) (mean ± SEM)	SM (n = 10) (mean ± SEM)	P Value
Has-miR-6087	1.287 ± 0.350	0.895 ± 0.529	0.544
Has-miR-451a	1.562 ± 0.344	1.780 ± 0.771	0.799
**Has-miR-4492**	1.155 ± 0.236	0.145 ± 0.037	**0.001**
**Has-miR-3960**	1.148 ± 0.215	0.476 ± 0.072	**0.008**
**Has-miR-4488**	1.240 ± 0.318	0.500 ± 0.105	**0.040**
Has-miR-144-3p	4.376 ± 2.443	4.492 ± 2.464	0.974
Has-miR-498-5p	1.421 ± 0.379	0.756 ± 0.515	0.312
Has-miR-512-3p	1.637 ± 0.482	1.196 ± 0.819	0.649
Has-miR-515-5p	1.819 ± 0.508	1.750 ± 1.144	0.956
**Has-miR-541-3p**	1.208 ± 0.257	0.226 ± 0.064	**0.002**
Has-miR-520 g-5p	1.228 ± 0.255	0.551 ± 0.320	0.116
Has-miR-592	2.433 ± 1.150	0.136 ± 0.047	0.061
**Has-miR-410-5p**	1.226 ± 0.335	0.090 ± 0.024	**0.003**
Has-miR-372-5p	1.918 ± 1.041	0.174 ± 0.043	0.112
**Has-miR-541-5p**	1.209 ± 0.252	0.272 ± 0.095	**0.003**

*NP* normal pregnant, *SM* Spontaneous miscarriages

The bold values represent the names and p-values of miRNAs that were statistically different in the NP and SM groups

### Plasma miR-410-5p levels were positively associated with early pregnancy and negatively correlated to the risk of miscarriage

After that, we collected plasma samples from the women with normal pregnancy, TA, or SM before the estimated gestational age of 12 weeks, and the pre-pregnancy group, and detected miR-410-5p levels. As indicated in Fig. 1, the plasma level of miR-410-5p increased significantly in the pregnancy group compared with the pre-pregnancy group (Fig. 1D). Additionally, lower miRNA levels were observed in the plasma of the TA and SM groups compared with those of the pregnancy group (Fig. 1D). ROC analysis and area under curve (AUC) were performed to verify the predictive value of plasma miR-410-5p for pregnancy and pregnancy complications. Our data revealed that plasma miR-410-5p levels had a good predictive significance for pregnancy (AUC = 0·802, 95% CI 0·677–0·927, P < 0·001, Fig. 1E) and SM (AUC = 0·788, 95% CI 0·665–0·910, P < 0·001, Fig. 1F). Moreover, miR-410-5p levels were a potent marker for the prognosis of TA (AUC = 0·762, 95% CI 0·642–0·882, P < 0·001, Fig. 1G) rather than a candidate biomarker for TA (AUC = 0·629, 95% CI 0·486–0·772, P = 0·083, Fig. 1H).

### miR-410-5p primarily originated from fetal trophoblasts

**Fig. 2:**
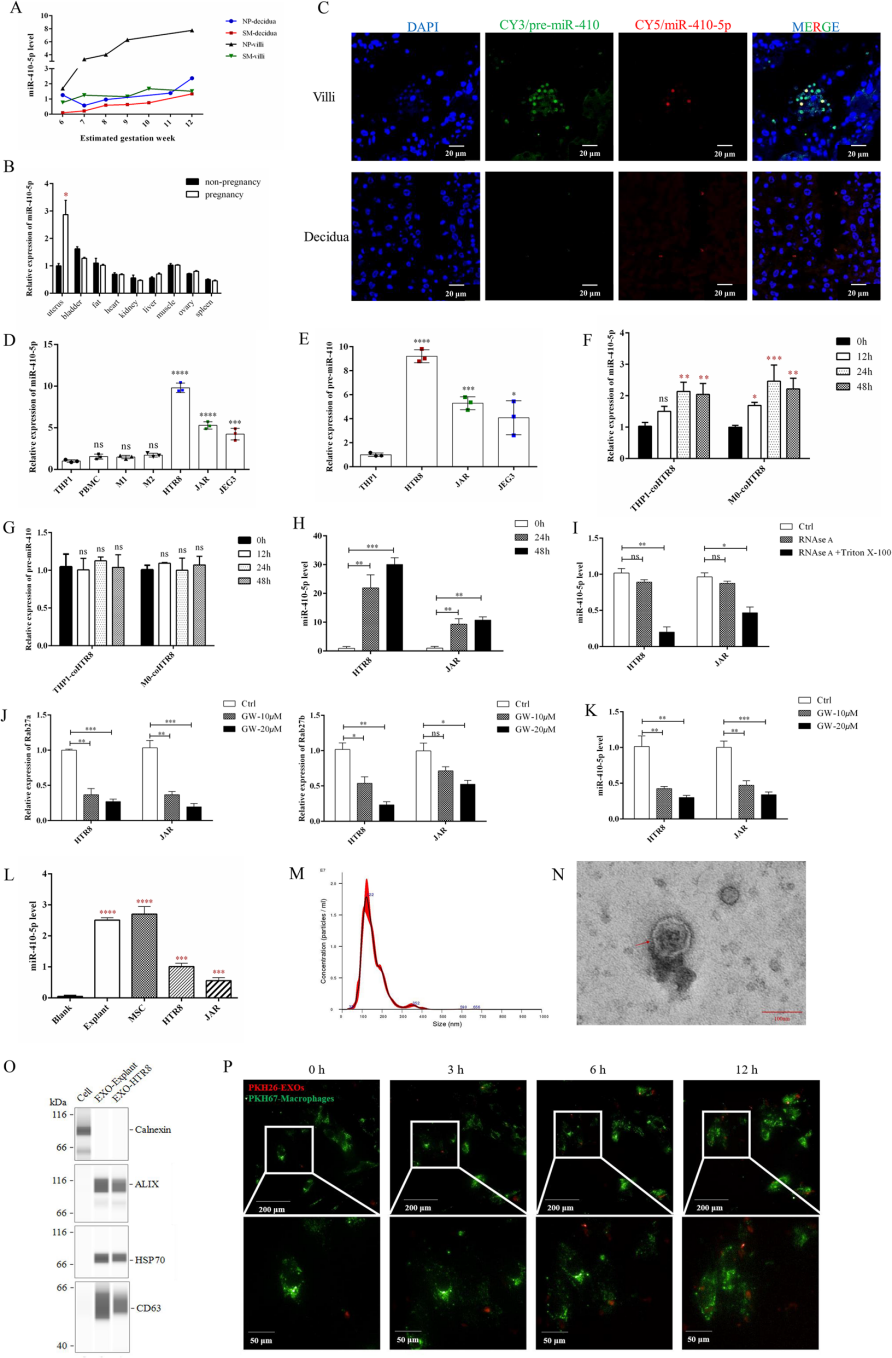
MiR-410-5p is transported from the fetal side to maternal side by EXOs. **A** qRT-PCR was used to verify the difference of miR-410-5p expression in induced abortion tissue and spontaneous abortion tissue in different gestational weeks. **B** The expression of miR-410-5p was analyzed using qRT-PCR in different organs of mice before and after pregnancy. **C** FISH was used to detect in situ expression of pre-miR-410 and miR-410-5p in human villi and decidua tissues. **D** qRT-PCR was used to detect the difference of miR-410-5p expression between human trophoblast cells (HTR8/SVneo, JAR, JEG3) and human monocyte cell (THP1), M1 macrophage, M2 macrophage and human peripheral blood mononuclear cells (PBMC). **E** The expression of pre-miR-410 in THP1 and human trophoblast cell lines were detected using qRT-PCR. **F–G** qRT-PCR was used to detect the expression of miR-410-5p and pre-miR-410in THP1 and M0 non-contact co-cultured with HTR8/SVneo for 12, 24 and 48 h. **H** qRT-PCR was used to detect the expression of miR-410-5p in the medium of HTR8/SVneo and JAR for 24 h and 48 h.** (I)** The medium of HTR8/SVneo and JAR was treated with RNase A alone or combined with Triton X-100 for 24 h, and the expression of miR-410-5p was detected busing qRT-PCR. **J–K** The Rab27a, Rab27b and miR-410-5p expressions were detected using qRT-PCR after HTR8/SVneo and JAR were treated with 10 μM and 20 μM GW4869 for 24 h. **L** qRT-PCR was used to detect the expression of miR-410-5p in placenta explants, mesenchymal stem cells (MSC), HTR8/SVneo and JAR cultured medium. **M** The particle size, distribution, and concentration of the EXOs were analyzed using NTA. **N** Transmission electron microscope images of representative EXOs with lipid bilayer structure. **O** The EXOs components and cell lysis products of primary placental explants and trophoblasts were analyzed by Simple Western with EXOs phenotypic protein antibodies (CD63, HSP70, ALIX) and cyto-protein Calnexin antibody. **P** PKH-67(green fluorescence) labelled macrophages and PKH-26(red fluorescence) labelled HTR8/SVneo EXOs. After the macrophages were treated with EXOs, the macrophages were photographed continuously for 12 h. Values were listed as the mean ± SD. *P < 0·05, **P < 0·01, ***P < 0·001, ****P < 0·0001

Subsequently, we investigated the origin of the miR-410-5p expression. As suggested in Fig. 1C, miR-410-5p levels in the fetal villi were much higher than those in the decidua from clinical samples. This phenomenon was consistent with the data from the mouse pregnancy model; that is, the levels of miR-410-5p in the pregnancy uterine decidua were significantly higher than those from the uterus of non-pregnant mice (Fig. 2B). Moreover, miR-410-5p levels gradually increased with elevated estimated gestation weeks (EGW) from EGW6 to EGW12 (Fig. 2A). Therefore, we postulated that miR-410-5p might originate from fetal trophoblasts. This hypothesis was further supported by an in vitro study. The levels of pre-miR-410 and miR-410-5p in trophoblast cells HTR8/SVneo, JAR, and JEG3 were all significantly higher than those in THP1, M1 macrophages, M2 macrophages, and peripheral blood mononuclear cells (PBMC) (Fig. 2D). Additionally, we noticed that pre-miR-410 (the precursor of miR-410-5p) was significantly higher in trophoblasts than in THP1 (Fig. 2E). Concurrently, miRNA FISH confirmed that the expression levels of pre-miR-410 and miR-410-5p in villi tissues were significantly higher than those in decidual tissues (Fig. 2C). Moreover, miR-410-5p was mainly secreted and expressed in fetus-related cells, including mesenchymal stem, embryonic stem, placental epithelial, and amniotic epithelial cells, rather than in other cells or tissues from a published dataset (https://fantom.gsc.riken.jp/5/suppl/De_Rie_et_al_2017/). These results suggested that miR-410-5p mainly originated from fetal cells including trophoblast cells.

### EXOs transported miR-410-5p from the fetal to maternal side

Using the in vitro co-culture system, we observed that the intracellular miR-410-5p levels in monocytes and macrophages were significantly increased after co-culture with HTR8/SVneo, compared with the level of the precursor of miR-410 (Fig. 2F, G). This observation suggests that miR-410-5p in monocytes or macrophages originated from trophoblasts rather than from its precursor miRNA.

MiR-410-5p levels in the culture medium of HTR8/Svneo and JAR trophoblasts significantly increased in a time-dependent manner after culturing with conditional medium for 24 h or 48 h (Fig. 2H). To verify that trophoblasts released miR-410-5p in a soluble way or EXOs, the conditional media were treated with RNase A in the presence or absence of Triton X-100. As indicated in Fig. 2I, the content of miR-410-5p in the medium was slightly decreased in the RNase A-only group, whereas the content of miR-410-5p was significantly decreased in the presence of Triton X-100. This result indicated that miR-410-5p was released in a membrane-surrounded structure in the cell medium. Additionally, pretreatment with the EXOs inhibitor GW4869 remarkably decreased the levels of miR-410-5p in the culture medium, accompanied by the inhibition of Rab27a and Rab27b expression in HTR8/Svneo trophoblasts (Fig. 2J–K). Similar results were observed in JAR cells (Fig. 2J–K). Furthermore, we collected fresh villi from pregnant women to culture placental explants to verify the above observations. As depicted in Fig. 2L, high concentrations of miR-410-5p were observed in the EXOs of primary placental explants and mesenchymal stem cells (MSCs). These findings suggest that miR-410-5p is mainly released by trophoblasts in the form of EXOs.

### EXO-miR-410-5p was captured by maternal macrophages

The EXOs from trophoblasts were isolated and characterized by particle size, morphology, and protein markers (Fig. 2M–O). The particle sizes of trophoblasts ranged from 72 to 180 nm, further verified using a transmission electron microscope. Three protein markers, including ALIX, HSP70, and CD63, were present in the EXOs, while Calnexin was absent. As shown in Fig. 2P, PKH26-labeled HTR8/Svneo-EXOs were engulfed into PKH67-labeled macrophages time-dependently. The above findings suggest that trophoblast cell-derived EXOs are captured by macrophages.

### miR-410-5p promoted in vitro M2 polarization

**Fig. 3:**
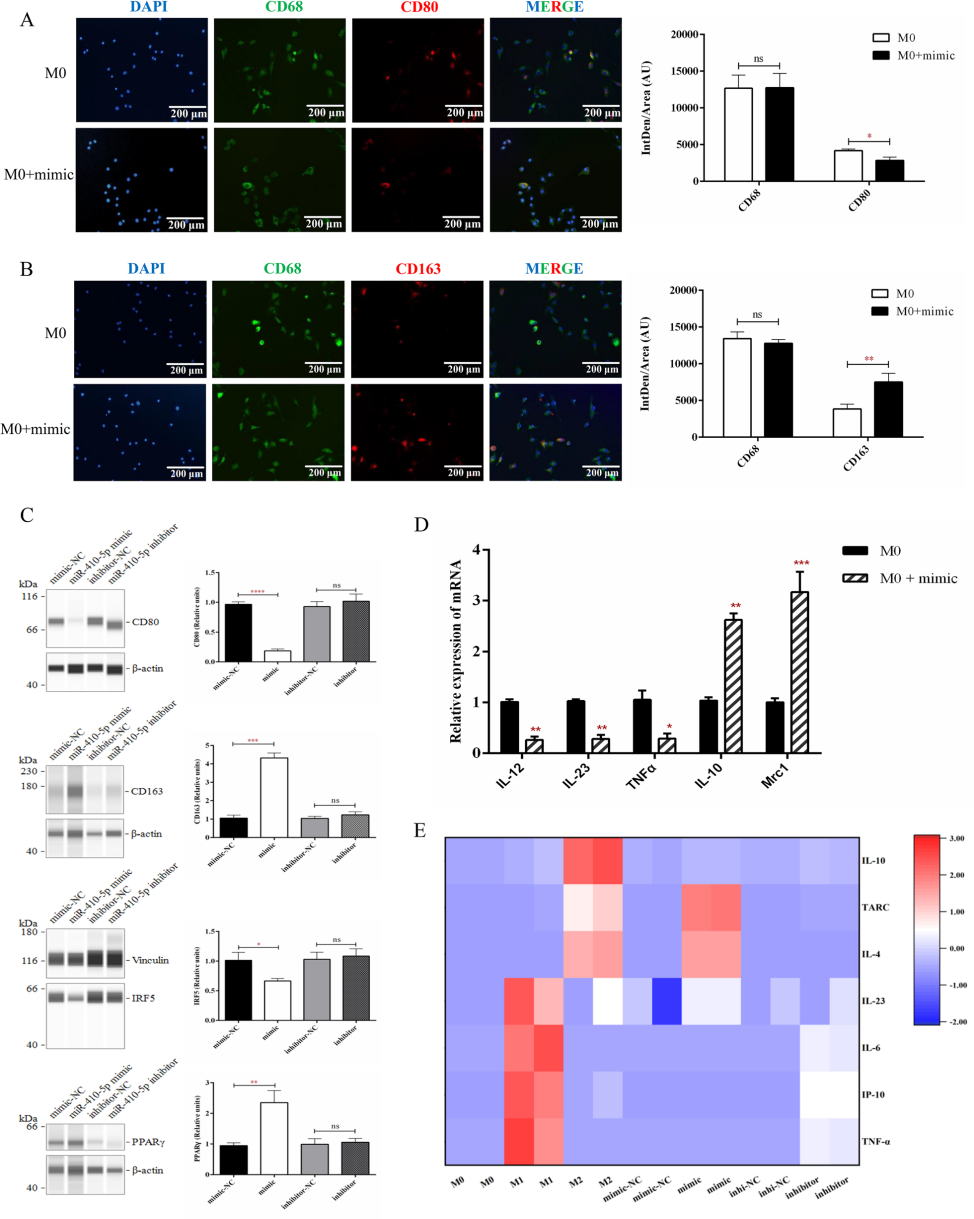
MiR-410-5p regulates macrophage phenotype. **A** The protein expressions of CD68 and CD80 were analyzed using immunofluorescence in M0 macrophages. **B** The protein expressions of CD68 and CD163 were analyzed using immunofluorescence in M0 macrophages. **C** The macrophages transfected with miR-410-5p mimic and inhibitor were analyzed by Simple Western with macrophage phenotypic antibodies (CD80, CD163, IRF5, and PPARγ). **D** The expression differences of IL-12, IL-23, TNF α, IL-10 and Mrc1 in M0 macrophages were detected using qRT-PCR. **E** Multi-factor flow microspheres were used to detect the expression of IL-4, IL-6, IL-10, IL23, IP-10, TARC and TNF α in the medium of M0/M1/M2 and transfected-M0 macrophages. Values were listed as the mean ± SD. ns, not significant, *P < 0·05, **P < 0·01, ***P < 0·001, ****P < 0·0001

To evaluate the effects of miR-410-5p on macrophage polarization, we successfully induced M0 macrophages from THP-1 monocytes with PMA, subsequently primed M0 towards M1 or M2 macrophages by LPS as well as IFN-γ or IL-4 plus IL-13, individually. Furthermore, immunofluorescent signals of CD163 were enhanced and signals of CD80 were decreased in M0 macrophages that were supplemented with miR-410-5p mimic (Fig. 3A, B). To further determine the effects of miR-410-5p on macrophage polarization, total proteins from M0 macrophages receiving miR-410-5p treatment were extracted and subjected to western blotting. The protein levels of the M2-related surface marker CD163 and transcriptional factor, peroxisome proliferator-activated receptor γ (PPARγ), were significantly elevated, accompanied by obviously inhibited protein levels of CD80, a M1-related surface marker, and interferon regulatory factor (IRF) 5, a transcriptional factor, in macrophages challenged with miR-410-5p (Fig. 3C). As depicted in Fig. 3D, anti-inflammatory signals, including IL-10 and mannose receptor C-type 1 (Mrc1) mRNAs, were significantly up-regulated, while pro-inflammatory signals containing IL-12, IL-23, and TNF-α mRNAs were remarkably downregulated in M0 macrophages treated with miR-410-5p mimic.

Subsequently, a multi-analyte flow assay kit with 7-plex macrophage-related cytokines was used to verify the effects of miR-410-5p on macrophage polarization. The results showed that M1 macrophages secreted high levels of pro-inflammatory factors, including IL-6, IL-23, interferon γ inducible protein-10 (IP-10), and TNF-α, and low levels of anti-inflammatory factors, such as IL-4, IL-10, and thymic and activating regulatory chemokine (TARC). A reversed cytokine pattern was observed in M2 macrophages. Interestingly, after treatment with miR-410-5p, M0 macrophages expressed a cytokine pattern similar to that of M2 rather than M1 macrophages (Fig. 3E), suggesting that miR-410-5p drives M0 macrophages to mature into anti-inflammatory M2 macrophages. Furthermore, the priming effects of miR-410-5p on macrophage polarization were markedly abolished by a specific inhibitor (Fig. 3E). Based on the above findings, we speculated that miR-410-5p may inhibit the M1 phenotype of macrophages and promote their polarization and maturation into M2 macrophages.

### STAT1 served as a target gene of miR-410-5p in macrophages

To identify the molecular mechanisms for priming macrophage polarization by miR-410-5p, a commercial 80-plex cytokine chip array and bulk RNA sequencing were performed in THP1-induced M0 macrophages transfected with miR-410-5p mimic. Data from the cytokine chip array indicated that there were a total of 33 differential proteins, including 13 downregulated proteins and 20 up-regulated proteins identified by fold change ≤  < 0·83 or fold change >  ≥ 1·2. Notably, several immune tolerance-related cytokines were up-regulated in M0 and M1 cells, such as IL-4, IL-13, macrophage-derived chemokine (MDC), transforming growth factor (TGF)-β2, vascular endothelial growth factor (VEGF), and osteopontin. GO analysis showed that most differential proteins were enriched in the JAK-STAT signaling pathway and cytokine and chemokine production (Additional file 1: Figure S1A). KEGG analysis also highlighted that genes of the JAK-STAT signaling pathway might be targets of miR-410-5p during macrophage polarization (Additional file 1: Figure S1B).

Bulk RNA sequencing showed that there were 212 DEGs, including 113 up-regulated genes and 99 down-regulated genes by |log2(Fold Change)|> 1 and P adj < 0·05, between M0 macrophages and cells challenged with miR-410-5p. Under the same screening conditions, 91 up-regulated and 18 down-regulated genes were also screened in M1 macrophages. The expression of phenotypic markers or immune tolerance-related chemokines of M2 macrophages, such as CD209 and CCL22, was activated by miR-410-5p. Consistent with the cytokine chip results, GO analysis of RNA sequencing data showed that DEGs were enriched in the JAK-STAT signaling pathway, cytokine, and chemokine production (Additional file 1: Figure S1C). KEGG analysis also highlighted that genes of the JAK-STAT signaling pathway might be targets of miR-410-5p during macrophage polarization (Additional file 1: Figure S1D).

The above findings suggested that JAK-STAT signaling might be the target for miR-410-5p in macrophages. Hence, we used three independent databases (miRDB, TargetScan, and DIANA-microT) to predict the possible targets of miR-410-5p (Additional file 1: Figure S1E). Additional file 1: Figure S1F shows the genes screened for common predicted targets in the three databases, which contain STAT1.

**Fig. 4:**
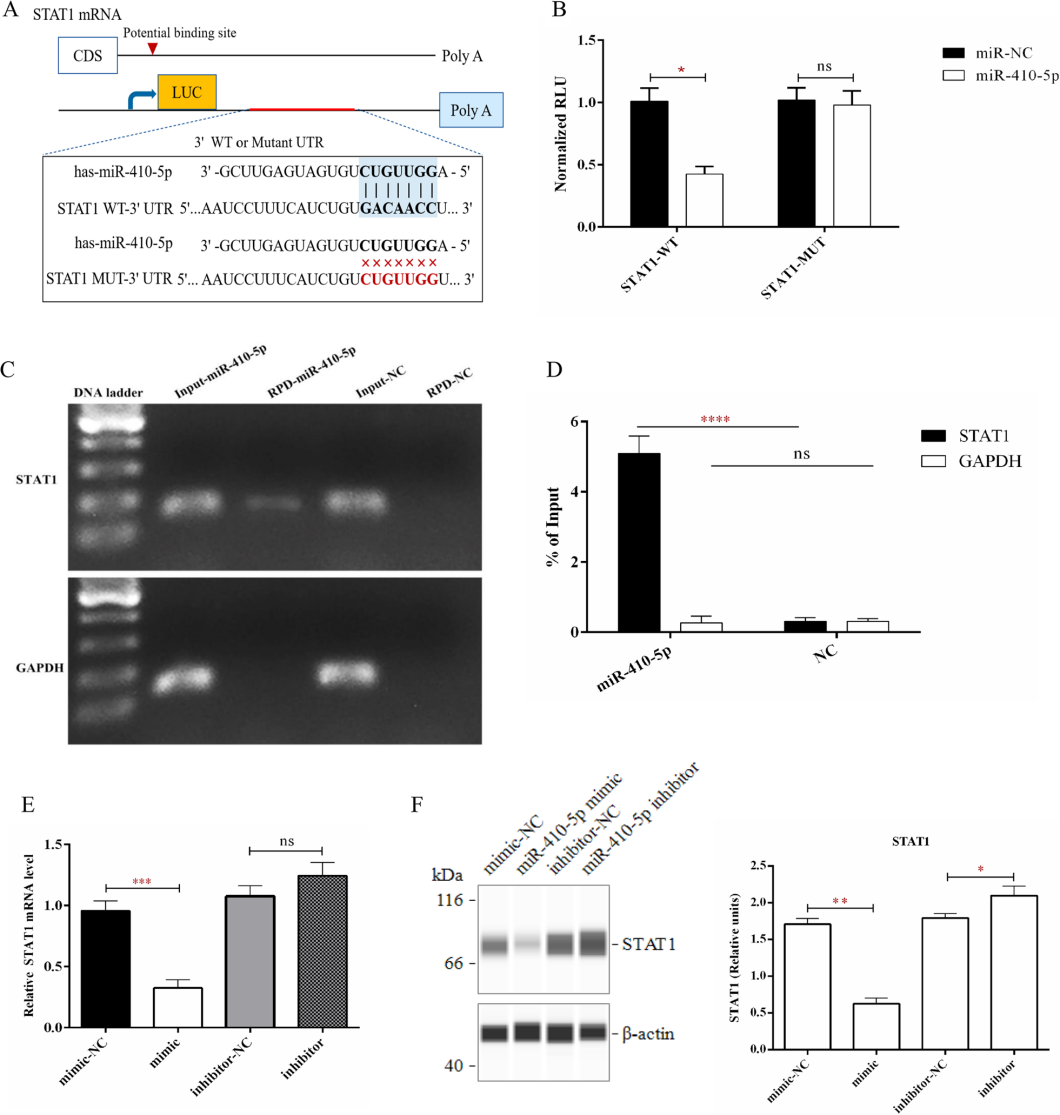
MiR-410-5p regulates STAT1 in vitro. **A** Schematic diagram of STAT1 non-coding regions and the mutation that occurred at the predicted miR-410-5p binding site. **B** The relative luciferase activity of macrophages transfected with WT-STAT1 + miR-NC, WT-STAT1 + miR-410-5p, MUT-STAT1 + miR-NC, and MUT-STAT1 + miR-410-5p. **C** Using PCR to verify the efficiency of miR-410-5p binding STAT1 RNA in miRNA pull-down experiment. **D** Using qRT-PCR to verify the efficiency of miR-410-5p binding STAT1 RNA in miRNA pull-down experiment. **E** The expression of STAT1 mRNA in macrophages transfected with miR-410-5p mimic or inhibitor was detected by qRT-PCR. **F** The expression of STAT1 protein in macrophages transfected with miR-410-5p mimic or inhibitor was detected by Simple Western. Values were listed as the mean ± SD. *Ns* not significant, *P < 0·05, ***P < 0·001, ****P < 0·0001

Dual-luciferase reporter and miRNA pull-down assays were used to determine whether STAT1 was the directly regulated target gene for miR-410-5p in macrophages. First, we constructed pmir-Luc-STAT1 WT and MUT vectors and transfected them into 293 T cells in the presence of a miR-410-5p mimic (Fig. 4A). We observed that miR-410-5p significantly reduced the luciferase activity of STAT1 WT 3'-UTR but did not affect the enzyme activity of MUT 3'-UTR (Fig. 4B). Subsequently, we constructed an miR-410-5p biotin probe and an NC probe and performed the miRNA-RNA pull-down assay with streptavidin magnetic beads. As indicated in Fig. 4C–D, results from PCR and qPCR confirmed that miR-410-5p was directly bound to STAT1 mRNA (~ 200 bp). Additionally, data from qPCR and Simple Western supported this finding, implying that the mRNA and protein levels of STAT1 in macrophages were significantly inhibited after transfection with the miR-410-5p mimic (Fig. 4E, F).

### miR-410-5p inhibited M1 macrophage polarization and regulated macrophage metabolism by suppressing the STAT1 signal pathway

**Fig. 5:**
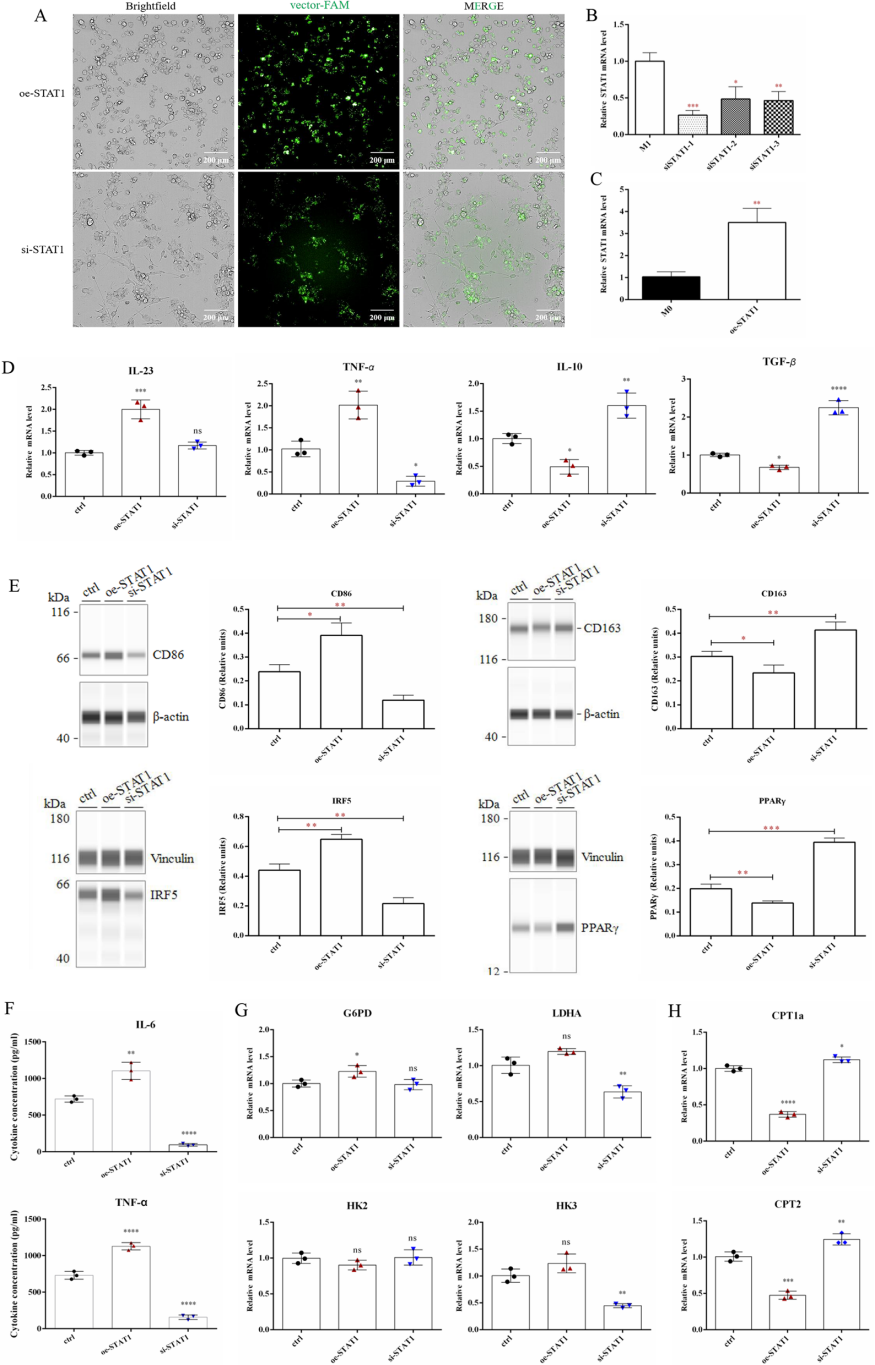
STAT1 activates macrophage M1 polarization. **A** The transfection efficiency of STAT1 overexpression vector(oe-STAT1) and interference vector(si-STAT1) in macrophages. **B** STAT1 knock-down efficiency in macrophages. **C** STAT1 overexpression efficiency in macrophages. **D** The expression differences of IL-23, TNF-α, IL-10 and TGF-β in induced-M0 macrophages by oe-STAT1 or si-STAT1 were detected by qRT-PCR. **E** The macrophages transfected with oe-STAT1 and si-STAT1 were analyzed by Simple Western with macrophage phenotypic antibodies (CD86, CD163, IRF5, and PPAR γ). **F** The contents of IL-6 and TNF α in the medium of macrophages were detected by ELISA. **G** The mRNA levels of glycolysis (G6PD, LDHA, HK2, HK3) in M0 macrophages were detected by qRT-PCR. **H** The mRNA levels of CPT1a and CPT2 in M0 macrophages were detected by qRT-PCR. (ctrl in D-H represents M0 macrophages). Values were listed as the mean ± SD. *Ns* not significant, *P < 0.05, **P < 0.01, ***P < 0.001, ****P < 0.0001

**Fig. 6:**
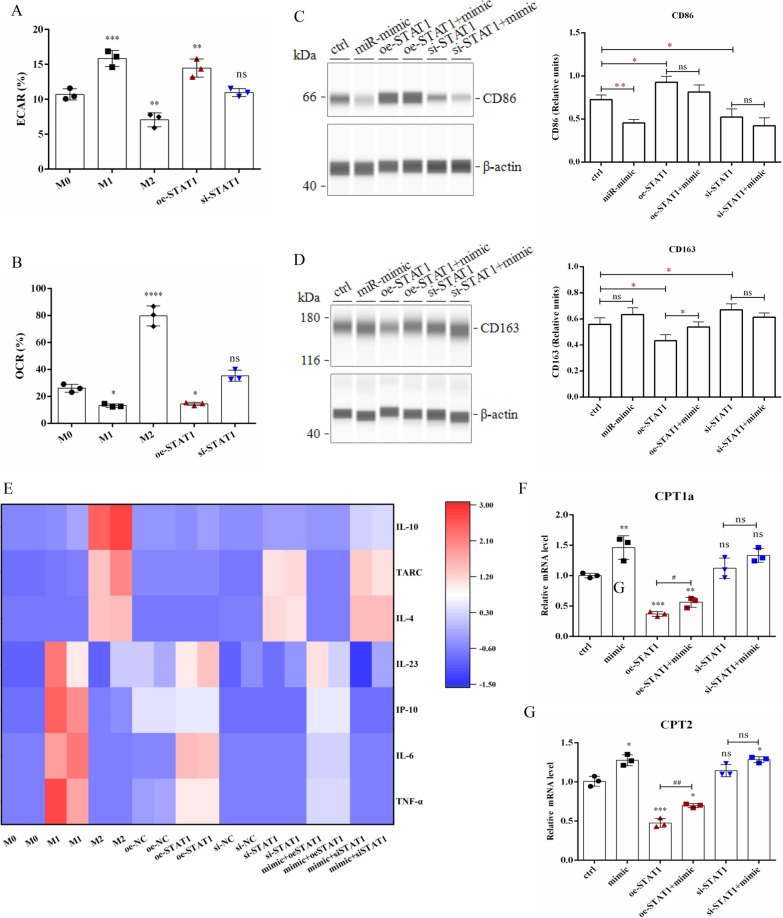
MiR-410-5p inhibits M1 macrophage polarization, regulates macrophage metabolism, and affects ROS generation by suppressing the STAT1 signal pathway. **A** The ECAR of M0/M1/M2 and transfected-M0 macrophages were detected using a multi-function enzyme labelling instrument. **B** The OCR of M0/M1/M2 and transfected-M0 macrophages were detected using a multi-function enzyme labelling instrument. **C** The macrophages transfected with miR-410-5p mimic, oe-STAT1 and si-STAT1 were analyzed by Simple Western with macrophage phenotypic antibodies CD86. **D** The macrophages transfected with miR-410-5p mimic, oe-STAT1 and si-STAT1 were analyzed by Simple Western with macrophage phenotypic antibodies CD163. **E** Multi-factor flow microspheres were used to detect the expression of IL-4, IL-6, IL-10, IL23, IP-10, TARC and TNF α in the medium of M0/M1/M2 and transfected-M0 macrophages. **F–G** The mRNA levels of CPT1a and CPT2 in M0 macrophages were detected using qRT-PCR. Values were listed as the mean ± SD. *ns* not significant, *P < 0·05, **P < 0·01, ***P < 0·001, ****P < 0·0001

To validate the function of STAT1 in macrophage polarization and metabolic alterations in this study, we constructed a STAT1 interference vector (si-STAT1) and overexpression vector (oe-STAT1) and intervened in the expression of STAT1 in macrophages (Fig. 5A–C). First, pro-inflammatory signals, including IL-23 and TNF-α mRNAs, were significantly up-regulated, while anti-inflammatory signals containing IL-10 and TGF-β mRNAs were remarkably down-regulated in oe-STAT1-induced macrophages (Fig. 5D). As depicted in Fig. 5E, the protein levels of CD163 and PPARγ were significantly inhibited, accompanied by elevated protein levels of CD86 and IRF5 in macrophages challenged with oe-STAT1. Opposite results were observed in si-STAT1-induced macrophages (Fig. 5D, E). ELISA data showed that oe-STAT1 significantly increased the secretion of IL-6 and TNF-α in macrophages, while si-STAT1 inhibited the secretion of these inflammatory factors (Fig. 5F). The above results suggest that STAT1 drives the maturation of macrophages into pro-inflammatory phenotypes. And this is well supported by existing literature [25–28]. To define the role of STAT1 in macrophage metabolism, we measured the expression levels of key enzymes involved in glycolysis and fatty acid metabolism. As shown in Fig. 5G, mRNA expression levels, including glucose-6-phosphate dehydrogenase (G6PD), hexokinase (HK)2, HK3, and lactate dehydrogenase A (LDHA), did not change significantly because of STAT1 intervention. However, the expression of carnitine palmitoyltransferase (CPT)1a and CPT2 mRNA in oe-STAT1-induced macrophages was significantly inhibited, while si-STAT1 slightly elevated their levels (Fig. 5H). Furthermore, we examined macrophage OCR and ECAR changes after intervention. M2 macrophages exhibited metabolic characteristics of high OCR and low ECAR, whereas M1 macrophages exhibited the opposite. This study showed that oe-STAT1 increased ECAR and reduced OCR levels in macrophages, even though si-STAT1 did not significantly alter M0 macrophage metabolism (Fig. 6A, B). Those data suggesting that mitochondrial fatty acid oxidation (FAO) may be an important metabolic pathway for STAT1 to regulate macrophage immune tolerance.

To further elucidate the role of miR-410-5p in regulating STAT1 during macrophage polarization, we treated M0 macrophages with oe-STAT1 or si-STA1 in combination with miR-410-5p mimic, respectively. Simple Western results show that miR-410-5p mimic could effectively reverse the effects of oe-STAT1 and enhance the effect of si-STAT1 on macrophages (Fig. 6C, D). Similarly, the multi-analyte flow assay indicated that after treatment with si-STAT1, macrophages expressed a cytokine pattern similar to that of M2 macrophages, while oe-STAT1-induced macrophages were more inclined toward M1 macrophages (Fig. 6E). After the addition of miR-410-5p mimic, the oe-STAT1-induced M1 phenotypic characteristics were weakened, which corresponded to the further enhancement of the si-STAT1-activated M2 phenotype (Fig. 6E).To further clarify the regulatory effect of STAT1 on FAO, we used an miR-410-5p mimic to attack macrophages modified with STAT1 and observed that the miR-410-5p mimic could significantly increase the mRNA levels of CPT1a and CPT2 in macrophages and effectively antagonize the inhibition of CPT1 and CPT2 by oe-STAT1 (Fig. 6F, G).

**Fig. 7:**
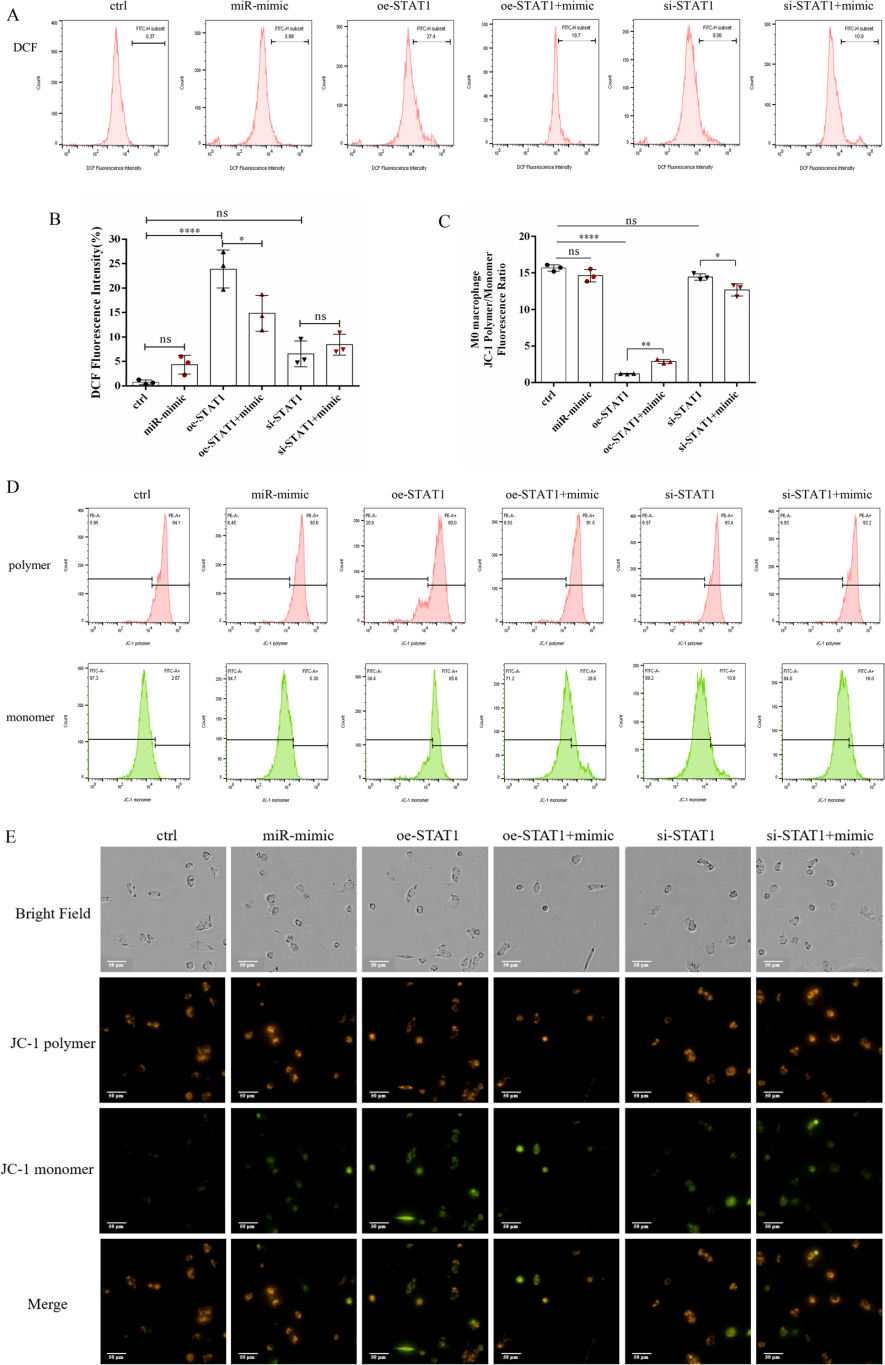
MiR-410-5p regulates MMP changes via STAT1 signaling in macrophages. **A** The level of ROS in M0 macrophages was detected by flow cytometry. **B** DCF probe fluorescence intensity (ROS level) statistics in macrophages. **C** Fluorescence intensity ratio of JC-1 polymer and monomer. **D** The fluorescence intensities of JC-1 polymers and monomers in macrophages was detected using flow cytometry.** E** The fluorescence image of JC-1 polymers and monomers in macrophages was detected using the Operetta CLS High connotative Analysis system. *ns* not significant, *P < 0·05, **P < 0·01, ****P < 0·0001

Finally, we simultaneously examined ROS levels in macrophages and MMP changes. As indicated in Fig. 7A, B, oe-STAT1 significantly promoted the production of ROS in M0 macrophages, and correspondingly, the miR-410-5p mimic and si-STAT1 did not exacerbate the accumulation of ROS. Importantly, the miR-410-5p mimic could inhibit the outbreak of ROS caused by oe-STAT1 (Fig. 7A, B). In M0 macrophages, after oe-STAT1 attacked macrophages, the fluorescence intensity of JC-1 polymer decreased, while the fluorescence intensity of JC-1 monomer increased significantly, and its ratio was significantly lower than that of the control group (Fig. 7C, D). This proved that overactivation of STAT1 led to a decrease in the MMP of macrophages. Furthermore, the miR-410-5p mimic could effectively inhibit the effect of oe-STAT1, thereby reducing the fluorescence intensity of JC-1 monomer in cell mitochondria (Fig. 7C–E), indicating that miR-410-5p can competitively silence STAT1 to prevent mitochondrial depolarization and protect macrophages from inflammatory damage-induced apoptosis.

These results indicate that the activation of STAT1 leads to mitochondrial dysfunction and excessive accumulation of ROS, which may be an important cause of macrophage immune dysregulation. Moreover, the STAT1-induced redox changes during immune-inflammatory responses were orchestrated by the actions of miR-410-5p, suggesting that the miR-410-5p/STAT1 signaling axis might play an important role in the functional and phenotypic regulation of decidual macrophages. However, presently, mining of key molecular signals is still relatively superficial, highlighting the need for future research in the field.

### miR-410-5p prevented pregnancy loss in LPS-induced miscarriage mice

**Fig. 8:**
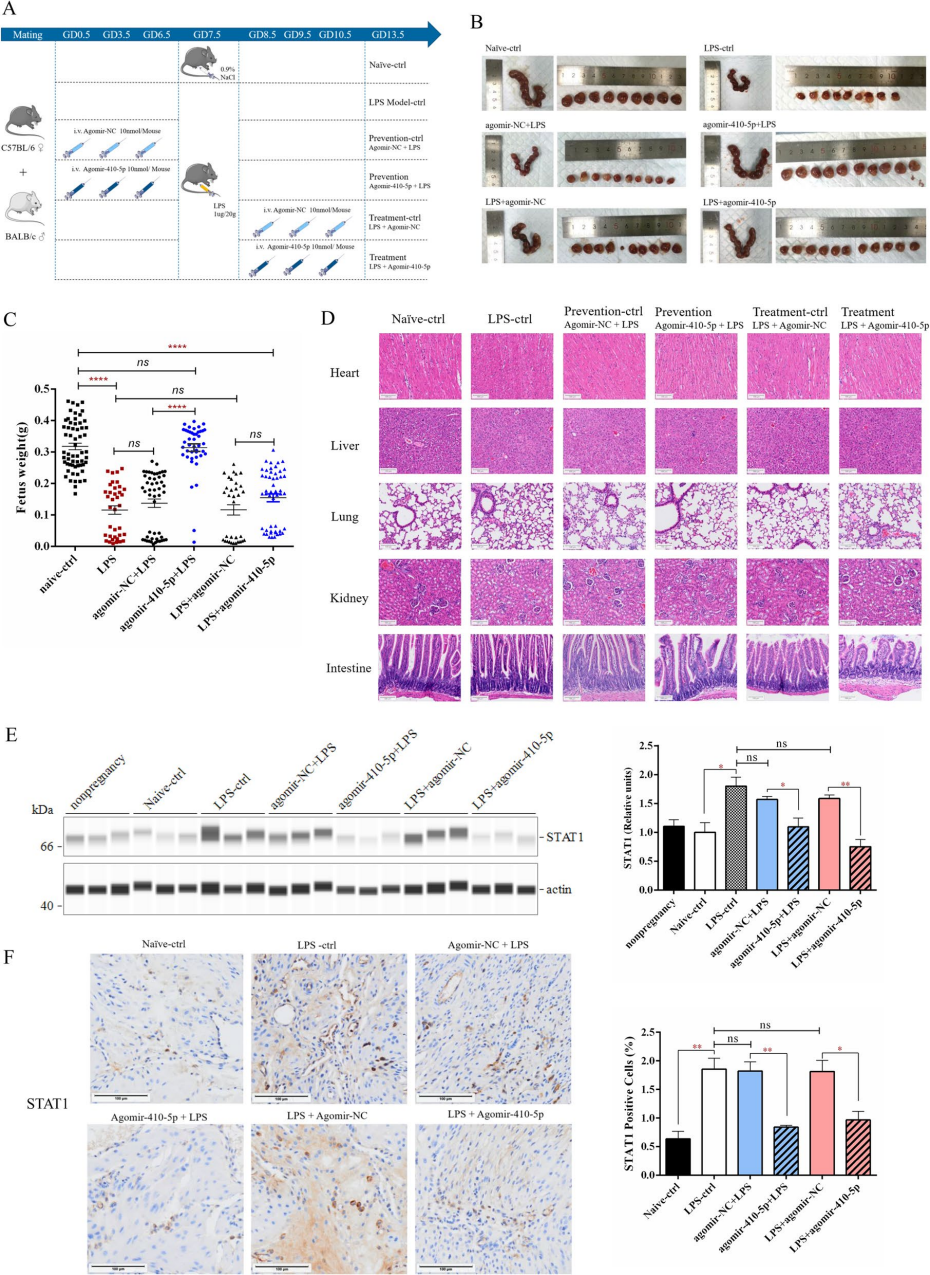
MiR-410-5p prevents LPS-induced abortion in mice. **A** Schematic diagram of animal experiment design and procedures. **B **Embryonic development of mice in each group on GD13·5 days. **C** The effect of agomir-410-5p on embryo quality before or after LPS infection. **D** HE staining was used to analyze the inflammatory injury of heart, liver, lung, kidney and small intestine in each group. **E** Simple Western was used to detect the expression of STAT1 proteins in the uterine tissue of mice (n = 3). **F** IHC was used to detect the expression and distribution of STAT1 proteins in the uterine tissue of mice (n = 3). Values were listed as the mean ± SEM. *Ns* not significant, * P < 0·05, **P < 0·01, ****P < 0·0001

To clarify the key role of miR-410-5p in pregnancy, we constructed normal pregnant mice and LPS abortion models for in vivo verification. For aborted mice, we established prevention and treatment groups according to the time of LPS infection. The prevention group was supplemented with three shots of agomir-410-5p before LPS infection on GD7·5, whereas the treatment group was supplemented with agomir-410-5p after LPS infection on GD7·5 (Fig. 8A). Histological examination showed that supplementation of exogenous miR-410-5p did not affect the tissue ischemia, edema, or necrosis of the heart, liver, lung, kidney, and intestine in mice (Fig. 8D). It could be clearly observed that pre-supplementation with miR-410-5p can effectively prevent LPS-mediated abortion and reduce embryo absorption (Fig. 8B, C). Unfortunately, there were few therapeutic effects of miR-410-5p on embryo absorption when LPS was pre-injected into pregnant mice (Fig. 8B, C). These results suggest that miR-410-5p is an important messenger between fetal trophoblasts and maternal decidual tissues at the maternal–fetal interface. Pretreatment with miR-410-5p may be an effective strategy to prevent inflammation-related abortion.

By comparing the sequences of mouse and human STAT1, we observed that the predicted sites of STAT1 targeted by miR-410-5p were highly consistent (Additional file 1: Figure S1G). Therefore, we further observed whether miR-410-5p had a targeted modulation effect on STAT1 in pregnant mice. It was confirmed that supplementation with miR-410-5p can significantly inhibit the expression of STAT1 protein in mouse decidua (Fig. 8E, F). These results suggest that STAT1 is a direct target of miR-410-5p, indicating that miR-410-5p binds to STAT1 and inhibits mRNA transcription and protein translation.

### miR-410-5p increased M2 macrophage polarization in miscarriage mice

**Fig. 9:**
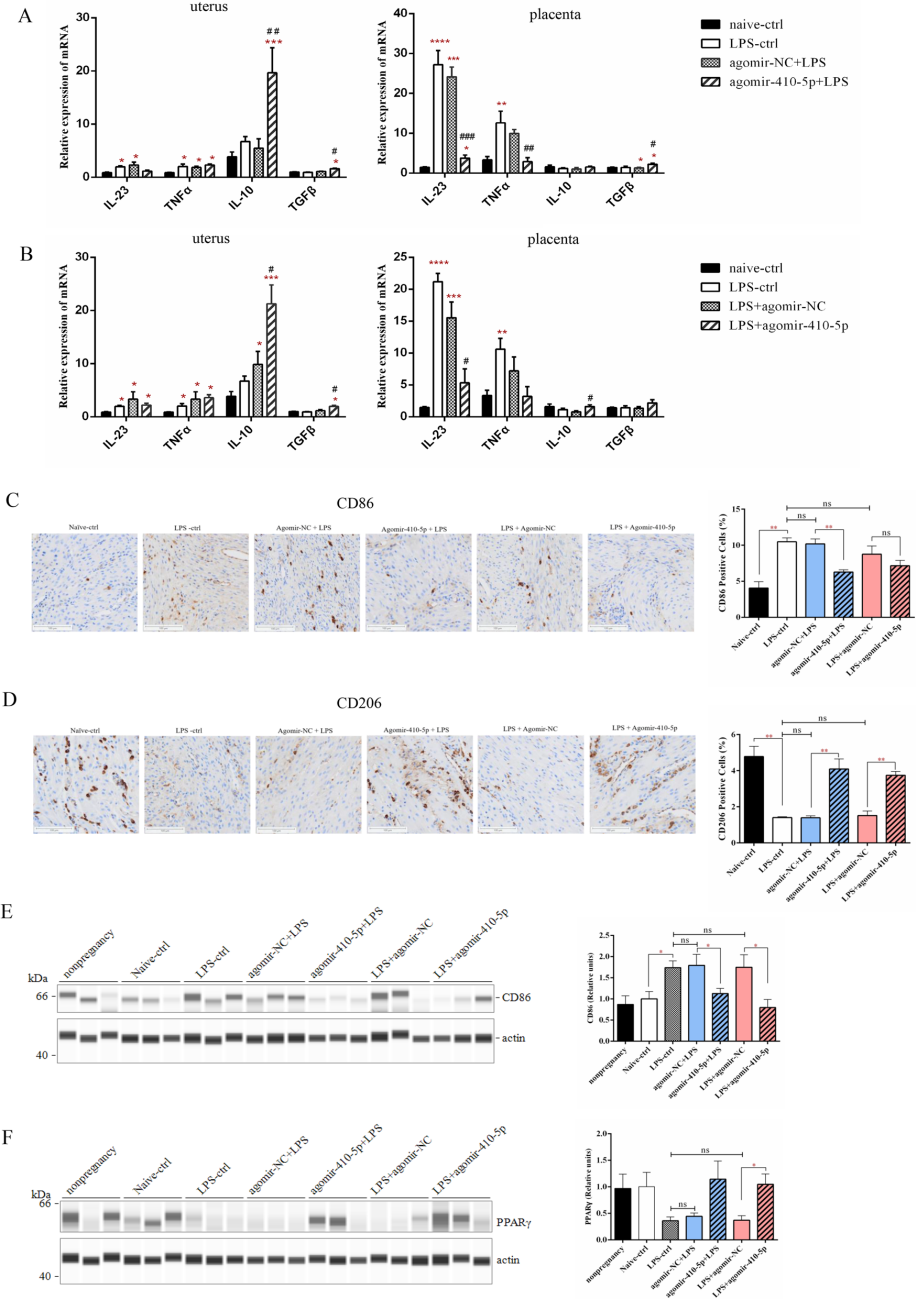
MiR-410-5p maintains maternal–fetal interface immune tolerance in vivo. **A** The expression differences of IL-23, TNF-α, IL-10, and TGF β in the pregnant uterus and placenta of mice supplemented with agomir-410-5p before LPS infection were detected using qRT-PCR. **B** qRT-PCR was used to detect the expression differences of IL-23, TNF-α, IL-10, and TGF β in the pregnant uterus and placenta of agomir-410-5p-supplemented mice after LPS infection. (* represents the difference in LPS- the related group compared with the naive-ctrl group, and ^#^ represents the difference between agomir-410-5p and agomir-NC). **C** IHC was used to detect the expression of CD86 proteins in the uterine tissue of mice (n = 3). **D** IHC was used to detect the expression of CD206 proteins in the uterine tissue of mice (n = 3). **E** Simple Western was used to detect the expression of CD86 proteins in the uterine tissue of mice (n = 3). **F** Simple Western was used to detect the expression of PPAR γ proteins in the uterine tissue of mice (n = 3). Values were listed as the mean ± SEM. *Ns* not significant, * P < 0·05, **P < 0·01, ***P < 0·001, ****P < 0·0001

Surprisingly, miR-410-5p at the maternal–fetal interface of mice was observed to have anti-inflammatory effects. During miR-410-5p prevention or treatment, varying degrees of downregulation of IL-23 and TNF-α were detected in the uterus and placenta of mice, accompanied by an increase in IL-10 or TGF-β (Fig. 9A, B). More importantly, miR-410-5p was found to effectively reduce the number and protein expression level of CD86^+^ macrophages, enhance the expression of PPAR protein, and significantly increase the proportion of CD206^+^ macrophages in the pregnant uterus of aborted mice treated with miR-410-5p (Fig. 9C–F). These findings further confirm that miR-410-5p inhibits the macrophage M1 phenotype and promotes its transition to the M2 phenotype.

## Discussion

This study discusses the importance of immune tolerance towards semi-allogeneic fetuses in ensuring successful human pregnancy. The maternal–fetal interface, which involves trophoblast, deciduous, and immune cells, plays a crucial role in this process [29, 30]. Decidual macrophages, the second most abundant number of decidual leukocytes in early pregnancy, maintain a phenotypic balance that supports pregnancy [31, 32]. M2 macrophage-mediated immune tolerance is essential for normal pregnancy; however, polarization and activation of macrophages towards M1-like phenotypes are associated with SM or RSA [16, 33]. Nevertheless, the mechanisms underlying decidual macrophage polarization in pregnancy and pregnancy-related diseases remain unclear.

**Fig. 10:**
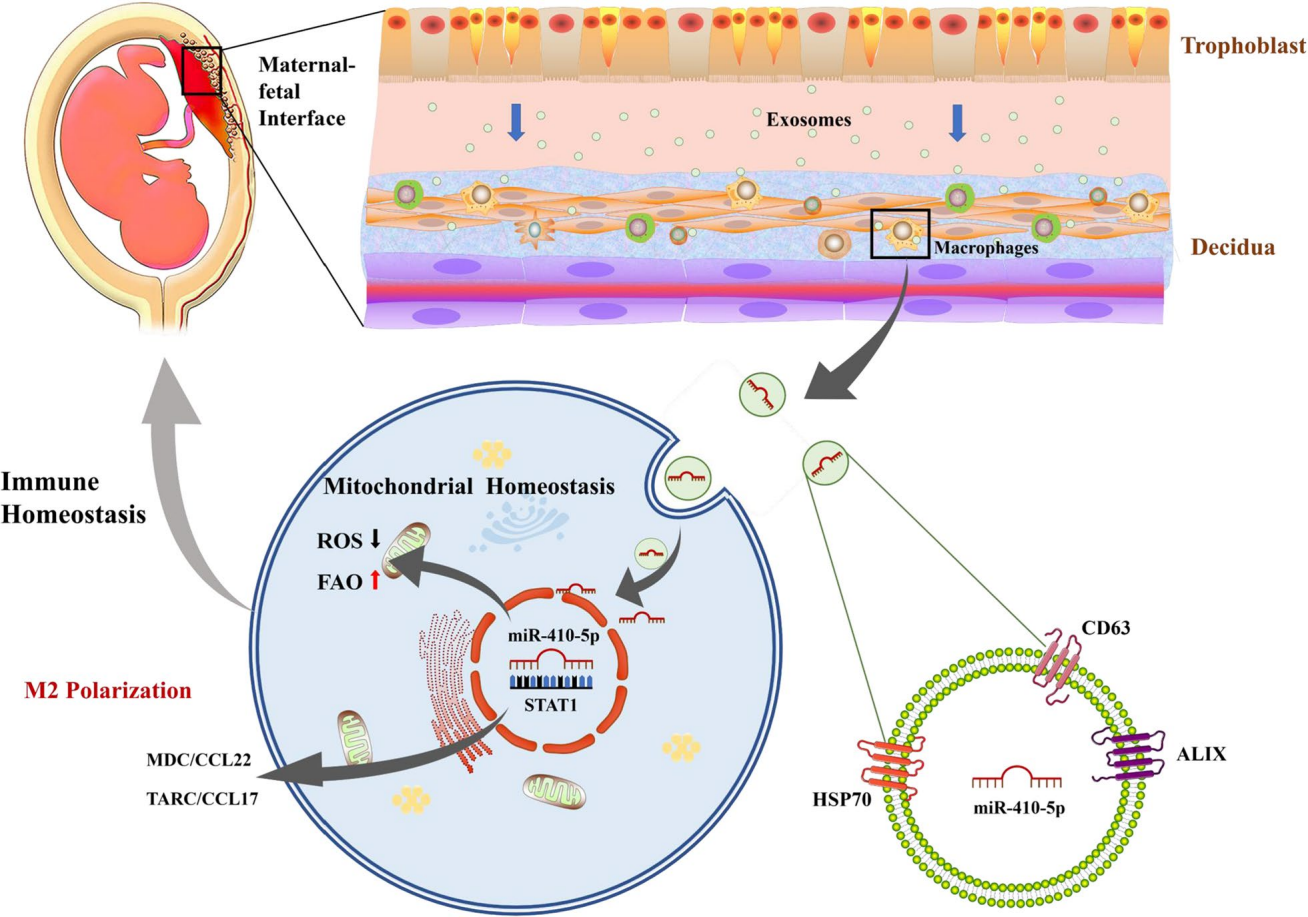
Schematic illustration of trophoblast-derived miR-410-5p inhibition of STAT1 regulating macrophage polarization at the fetal-maternal interface. Trophoblast-derived EXOs-miR-410-5p purposefully inhibits the intracellular activation of STAT1, reduces the accumulation of ROS, and prevents mitochondrial depolarization. Simultaneously, miR-410-5p further promoted FAO in cells, increased the secretion of MDC and TARC, ultimately induced M2 polarization, and participated in the maintenance of maternal–fetal interface immune homeostasis

Herein, we revealed that trophoblast-derived EXOs carried abundant miR-410-5p to the maternal interface and induced maternal immunosuppression by regulating macrophage polarization and function. More importantly, we provided novel evidence to suggest that trophoblast-derived EXO miR-410-5p plays an important role in M2 macrophage polarization by inhibiting the STAT1 signaling pathway. As shown in Fig. 10, miR-410-5p purposefully inhibited the activation of intracellular STAT1, reduced the accumulation of ROS, and prevented mitochondrial depolarization. Concurrently, miR-410-5p further promoted FAO in cells and increased the secretion of MDC and TARC, which ultimately induced M2 polarization (Fig. 10). Moreover, this study supports that exogenous supplementation with miR-410-5p can prevent LPS-induced miscarriage by inducing M2 macrophage polarization. Establishing maternal–fetal interface immune tolerance via miR-410-5p-related mechanisms may be an option for cell-free therapy for SM.

MiR-410 is located in the 14q32·31 region of the human chromosome and the 12F1 region of the mouse chromosome. After the primary miR-410 is processed in the nucleus, the precursor miR-410 is cleaved and matured into miR-410-3p and miR-410-5p, respectively. To date, miR-410-3p has been observed to be abnormally expressed in a variety of disorders, including cancers, inflammation, and neurodegenerative diseases, which involve diverse biological activities, such as cell proliferation, apoptosis, cell invasion, and drug resistance [34–36]. However, there are limited reports regarding the expression and biological actions of miR-410-5p, except in prostate cancer and myocardial fibrosis-related diseases. It has been reported that miR-410-5p, which can be secreted in tumor cells, adipose tissue, myocardial tissue, and kidneys, targets the degradation of mRNA or miRNA through base pairing, thereby participating in prostate cancer progression, myocardial fibrosis, and diabetic cardiomyopathy [37–39]. Our team performed small RNA sequencing on decidual CD14^+^ cells from three RSA patients and three normal pregnancy patients, and found that miR-410-5p was significantly downregulated in RSA. This increased our interest in the function and role of miR-410-5p in the maternal–fetal interface. Our previous study also showed that miR-410-5p in the placenta mediated trophoblast cell proliferation, apoptosis, invasion, and migration by regulating the expression of ITGA6 [40]. However, in the present study, we reported for the first time that miR-410-5p levels in villi and decidua from patients with SM were lower than those in patients that had experienced normal pregnancy and opted for an induced abortion. Quantitative analysis of serum and plasma samples confirmed that the content of miR-410-5p in peripheral blood increased after pregnancy, and the expression of circulating miR-410-5p decreased during spontaneous abortion. In addition, in the LPS-induced miscarriage mouse model, we discovered that intravenous injection of agomir-410-5p can reduce embryo absorption induced by the LPS challenge. Based on the above findings, we verified that high systemic or local levels of miR-410-5p after pregnancy are beneficial for the maintenance of pregnancy. Given the heterogeneity of clinical specimens and the incomplete detection techniques and means, we consider the bias in the study results to be understandable.

Furthermore, we observed that miR-410-5p levels in villi were significantly higher than those in decidual tissues. The levels of miR-410-5p in trophoblast cell lines were significantly higher than those in human PBMC and THP-1 monocytes. Consistent with our results, a published database supports the conclusion that miR-410-5p is mainly expressed in placental epithelial cells, though its expression is low in monocytes and macrophages [41]. These results suggested that miR-410-5p is mainly secreted and released by trophoblasts during pregnancy. In addition, sheep embryonic RNA sequencing has shown that mir-410-5p is involved in fetal skeletal development, and its expression abundance is related to gestational age; a finding which comparatively supports mir-410-5p being considered fetal-derived miRNA [42]. In the initial screening, we found that miR-410-5p levels in decidual macrophages of the aborted uterus were markedly downregulated compared with those derived from a normal pregnancy patient. The origin of miR-410-5p in macrophages was one of our unanswered questions. Questions such as ‘How is miR-410-5p transported into macrophages?’ and ‘What role does miR-410-5p play during pregnancy?’ were the key problems our present study attempted to solve regarding the origin and function of miR-410-5p in pregnancy.

Interactive crosstalk between cells forms the basis of placental microenvironmental immune tolerance. Studies have confirmed that trophoblasts can regulate macrophage polarization and affect the maternal–fetal immune microenvironment through cytokines and soluble proteins [43]. Recently, researchers have speculated that EXOs are important messengers in intercellular communication, which has raised concerns. During pregnancy, placental EXOs are mainly synthesized by syncytiotrophoblast cells through the lysosomal pathway and released into the maternal circulation [17]. The level of EXOs in the peripheral blood of pregnant women was 13·2 times higher than that of non-pregnant women, and the concentration of placental-derived EVs in maternal plasma increased with the progression of pregnancy [44, 45]. More importantly, EXOs can regulate the function of lymphocytes and monocytes at the maternal–fetal interface to induce immune tolerance [17, 46, 47]. In this study, we cultured HTR8/SVneo and JAR, as well as placental explants of early pregnancy under hypoxia, and isolated EXOs by differential ultracentrifugation. EXOs from placental explants and trophoblast cells were rich in miR-410-5p. However, the amount of placental tissue obtained from early pregnancy patients in the study was less than that of the other groups, and did not meet the needs of the experiment; therefore, there is need for a follow-up experimental study which depends on trophoblast cells HTR8/SVneo and JAR.

miRNAs are a group of small non-coding RNAs that regulate gene expression by binding to the 3'-UTR of its target gene in a sequence-specific manner, thereby reducing gene expression [48]. Recent studies have shown that miRNAs, as special media involved in the regulation of cell communication, can participate in maternal–fetal crosstalk to maintain pregnancy [49, 50]. Previous studies by our team have shown that M1 macrophage-derived miR-146a-5p and miR-146b-5p directly inhibit the expression of tumor necrosis factor receptor-associated factor 6 (TRAF6) in trophoblasts [20]. This process inhibits the epithelial-mesenchymal transition of trophoblast cells, thereby reducing their ability to migrate and invade, ultimately leading to recurrent miscarriages [20]. Additionally, in pregnancy complications, such as preeclampsia, premature rupture of membranes, and recurrent abortion, miR-181c, miR-let-7b, miR-103, miR-100-5p, and miR-155 are involved in the regulation of decidual macrophage function and affect the phenotypic shift of macrophages according to changes in the placental immune microenvironment [51–55]. Although it has been proven that the abnormal expression of miRNAs may regulate the polarization of macrophages, the transport pathway and specific regulatory mechanism of miRNAs at the maternal–fetal interface remain unknown. In this study, we speculated from cytokine microarray and mRNA sequencing results that the regulation of miR-410-5p on macrophage polarization may be complex. For example, miR-410-5p can up-regulate the expression of IL-4, IL-13, VEGF, and osteopontin in macrophages; however, it is also accompanied by the activation of TNF-α. This may also be related to the dual regulation of osteopontin. Osteopontin not only enhances the phagocytosis of macrophages and maintains immune homeostasis but also participates in the recruitment of monocytes/macrophages and mediates the secretion of cytokines in leukocytes [56, 57]. Therefore, examining cytokine chips and mRNA sequencing showed that miR-410-5p could significantly increase the anti-inflammatory chemokines MDC/CCL22 and TARC/CCL17 expression, causing macrophages to shift towards the M2 phenotype. Collectively, the presence of miR-410-5p during polarization changes the expression of M1/M2-related markers and increases the production of immune tolerance-related cytokines. It is credible to suggest that miR-410-5p can promote the M2 polarization of macrophages.

Our study aimed to reveal the potential function of trophoblast-derived miRNAs in macrophage polarization during pregnancy, and we found that miR-410-5p downregulation through STAT1 signal-mediated M1 macrophage polarization may be a crucial cause of SM. M2 polarization of macrophages mediated by miR-410-5p is a significant pathway for maintaining normal pregnancy.

STAT1 is a member of the STAT family encoded by STAT1 on human chromosome 2q32·2 [58]. The STAT1 signaling pathway plays a vital role in M1 polarization. There is clear evidence that activation of STAT1 supports macrophage polarization to an ‘‘M1’’ state, characterized by increased pro-inflammatory activity and resistance to anti-inflammatory factors [25–27, 59]. Furthermore, STAT1 may also be involved in macrophage redox and metabolic regulation [60–63]. However, the role and potential mechanism of STAT1 in up-regulating M1 polarization in SM or RSA remain largely unknown. Our study showed that STAT1 may be a target of miR-410-5p through sequence prediction. Therefore, we speculated that the decrease in miR-410-5p may participate in spontaneous abortion by inhibiting the STAT1 signaling pathway to promote M1 polarization. Our results demonstrated that miR-410-5p reduces the expression of STAT1 by directly interacting with its 3’-UTR, whereas miR-410-5p inhibition shows the opposite result. Moreover, overexpression of STAT1 reversed the inhibitory effect of miR-410-5p on STAT1 expression and eliminated the inhibitory effect of miR-410-5p on M1 macrophage polarization. These results suggest that miR-410-5p negatively regulates M1 polarization via the STAT1 signaling pathway. In this study, we found that macrophage phenotypic alterations affect OCR and ECAR, and further determined the gene expression of key enzymes involved in glucose metabolism and oxidative phosphorylation. G6PD is the only rate-limiting enzyme in the pentose phosphate pathway (PPP), and rapidly proliferating cells require metabolites from PPP to synthesize ribonucleotides and maintain intracellular redox homeostasis [64, 65]. Both HK2 and HK3 can phosphorylate glucose to produce glucose-6-phosphate, which is the first step in most glucose metabolic pathways and is associated with an increase in the rate of glycolysis [66–68]. LDHA is one of the key enzymes in aerobic glycolysis and catalyzes the final step of glycolysis, converting pyruvate to lactic acid [69, 70]. CPT1a and CPT2 are the rate-limiting steps of catalytic FAO, the deficiency of which leads to a decrease in the rate of fatty acid β oxidation, while the high expression of CPT1a helps to restore mitochondrial homeostasis [71–73]. This study finds that STAT1 may inhibit FAO and induce ROS production, leading to mitochondrial dysfunction and disruption of immune tolerance in macrophages. ROS are important mediators for activating pro-inflammatory signaling pathways, which regulate the polarization and metabolic reprogramming of macrophages and affect their immune tolerance [74]. Whereas, MMP stability is a prerequisite for maintaining oxidative phosphorylation of mitochondria and is essential for regulating macrophage function [75]. During mitochondrial oxidative phosphorylation, MMP begins to decline in the early stages of apoptosis, indicating mitochondrial dysfunction, and the cyanine dye JC-1 facilitates discrimination of energized and deenergized mitochondria [75–77]. In this study, miR-410-5p prevented the excessive accumulation of ROS caused by STAT1 while inhibiting mitochondrial depolarization, thereby reducing apoptosis due to inflammatory damage in macrophages. This process may also be the important mechanism by which miR-410-5p regulates macrophage tolerance and maintains pregnancy at the maternal–fetal interface. The in vivo study further demonstrated that LPS infection activated the expression of STAT1 and promoted the polarization of M1 to participate in spontaneous abortion. Overexpression of miR-410-5p can inhibit M1 polarization by inhibiting STAT1, thereby effectively reducing embryo loss.

Recent studies have shown that miRNAs in the peripheral blood of pregnant women have the potential to be utilized as biomarkers for the early detection and treatment of targeted diseases. Promising biomarkers, such as miR-100-5p, miR-378d, and miR-215-5p, have been identified in early ectopic pregnancy [78, 79]. Additionally, our study revealed that the expression of miR-410-5p in the plasma of women with threatened abortion and spontaneous abortion decreased significantly. ROC analysis showed that miR-410-5p could sensitively distinguish between patients with spontaneous abortion and those with threatened abortion, indicating its potential as a diagnostic marker for SM. These findings provide valuable insights into the diagnosis, early detection, and treatment strategies for targeted diseases in pregnant women. However, an increase in miR-410-5p is also associated with some cancers. For example, plasma miR-410-5p is considered a specific marker for prostate cancer [80]. This suggests that when predicting pregnancy or miscarriage in the future, attention should be paid to excluding confounding factors. In addition, it should be noted that when miR-410-5p is applied in clinical treatment, it may induce tumors or aggravate their progression.

Our findings suggest that miR-410-5p, derived from trophoblasts, is crucial in regulating maternal–fetal tolerance. Through the STAT1 signaling pathway, miR-410-5p impacted the polarization of M2 macrophages. Furthermore, abnormal downregulation of miR-410-5p may hinder the STAT1 signaling pathway, leading to increased polarization of M1 macrophages and consequently contributing to spontaneous abortion. These results indicated that miR-410-5p could potentially serve as a diagnostic marker and therapeutic target for spontaneous abortion. However, it is important to note that the mechanisms underlying the miRNA regulation of immune tolerance at the maternal–fetal interface are diverse and may involve different target genes and binding sites. Further research is needed to fully understand the interaction network and crosstalk between miRNAs in the maternal–fetal interactive response.

## Conclusion

This study revealed that trophoblast-derived miR-410-5p is crucial in maintaining maternal homeostasis and immunotolerance towards semi-allograft fetuses during early pregnancy. This microRNA promotes M2 macrophage polarization by targeting STAT1, thereby ensuring a healthy pregnancy. These findings have significant implications for the diagnosis and prevention of early miscarriages, which provides a new perspective on the fetal-maternal interface and offers potential avenues for further research in this field. The importance of miR-410-5p in maintaining a successful pregnancy requires continued investigation to fully elucidate its mechanisms and potential therapeutic applications.

### Supplementary Information

**Additional file 1: Table S1.** The information of the reagents and chemicals. **Table S2.** The information of antibodies for protein simple wes. **Table S3.** Oligonucleotides. **Table S4.** The Sequences of Primers for Quantitative RT-PCR. **Table S5.** Summary of Reagents for Immunofluorescence. **Table S6.** The Minimum and Maximum Detectable Concentration of Cytokines. **Table S7.** Summary of Primary Antibodies and Immunohistochemical Technique. **Figure S1.** STAT1 is a potential target for miR-410-5p.

## Data Availability

All data needed to evaluate the conclusions in the paper are presented in the paper and/or in the supplemental information. Additional data related to this study may be requested from the authors. The datasets presented in this study can be found in online repositories. The names of the repository/repositories and accession number(s) can be found below: https://ngdc.cncb.ac.cn, PRJCA019099.
