# Supplementary material for: Trophoblast-derived miR-410-5p induces M2 macrophage polarization and mediates immunotolerance at the fetal-maternal interface by targeting the STAT1 signaling pathway
Source: J Transl Med. 2024 Jan 4;22:19. doi: 10.1186/s12967-023-04831-y (PMC10768263; doi:10.1186/s12967-023-04831-y)
Supplement: Supplementary file 1 — Additional file 1: Table S1. The information of the reagents and chemicals. Table S2. The information of antibodies for protein simple wes. Table S3. Oligonucleotides. Table S4. The Sequences of Primers for Quantitative RT-PCR. Table S5. Summary of Reagents for Immunofluorescence. Table S6. The Minimum and Maximum Detectable Concentration of Cytokines. Table S7. Summary of Primary Antibodies and Immunohistochemical Technique. Figure S1. STAT1 is a potential target for miR-410-5p. [file 12967_2023_4831_MOESM1_ESM.docx]

**Additional file Table**

**Table S1. The Information of the Reagents and Chemicals**

| **Reagents / Chemicals** | **Manufacturer** | **Product No.** | **City** | **Country** |
| --- | --- | --- | --- | --- |
| BCA assay kit | Beyotime | P0010 | Shanghai | China |
| BersinBio^TM^ Fluorescent in situ hybridization（FISH）Kit | BersinBio | Bes1001 | Guangzhou | China |
| BersinBio^TM^ miRNA pulldown Kit | BersinBio | Bes5108 | Guangzhou | China |
| DAPI solution | Beyotime | P0131 | Shanghai | China |
| DMEM medium | Procell | PM150210 | Wuhan | China |
| DMEM-F12 medium | Gibco | 11320033 | New York | USA |
| Dual-Luciferase® Reporter Assay System | Promega | E1910 | Madison | USA |
| Extracellular oxygen consumption assay | Abcam | Ab197243 | Massachusetts | USA |
| Fetal bovine serum (FBS) | Gibco | 10091148 | New York | USA |
| Glycolysis Assay [Extracellular acidification] | Abcam | Ab197244 | Massachusetts | USA |
| GW4869 | MCE | HY-19363 | New Jersey | USA |
| Human Cytokine G5 Antibody array | RayBiotech | AAH-CYT-G5 | Atlanta | USA |
| Human IL-6 ELISA Kit | Beyotime | PI325 | Shanghai | China |
| HumanTNF-α ELISA kit | Beyotime | PT518 | Shanghai | China |
| Lipopolysaccharides from Escherichia coli O111:B4(LPS) | Sigma-Aldrich | L4391 | Hessen Land | Germany |
| Liposome 2000 | Invitgen | 11668500 | California | USA |
| Luna® Universal qPCR Master Mix | NEB | M3003L | Massachusetts | USA |
| MEM medium | Procell | PM150410 | Wuhan | China |
| miRcute serum plasma miRNA extraction and separation kit | TIANGEN | DP503 | Beijing | China |
| miRNA 1st Strand cDNA Synthesis Kit | Accurate Biology | AG11717 | Changsha | China |
| miRNA 1st Strand cDNA Synthesis Kit (by stem-loop) | Vazyme | MR101 | Nanjing | China |
| miRNA Universal SYBR® qPCR  Master Mix | Vazyme | MQ101 | Nanjing | China |
| Mitochondrial membrane potential assay kit with JC-1 | Beyotime | C2006 | Shanghai | China |
| Multi-Analyte Flow Assay Kit of Macrophage/Microglia | BioLegend | 740503 | San Diego | USA |
| Penicillin-streptomycin | Gibco | 15140122 | New York | USA |
| Penicillin-Streptomycin-Amphotericin B | ATCC | PCS-999-002 | Virginia | USA |
| Phosphate buffer saline (PBS) | Gibco | 10010023 | New York | USA |
| Phospholipid 12-myristic acid 13-acetate (PMA) | Sigma-Aldrich | 79346 | Hessen Land | Germany |
| PKH26 Red Fluorescent Cell Linker Mini Kit for General Cell Membrane Labeling | Sigma-Aldrich | MINI26 | Hessen Land | Germany |
| PKH67 Green Fluorescent Cell Linker Mini Kit for General Cell Membrane Labeling | Sigma-Aldrich | MINI67 | Hessen Land | Germany |
| PrimeScript™ RT reagent Kit (Perfect Real Time) | Takara Bio | RR037Q | Beijing | China |
| Radioimmunoprecipitation (RIPA) | Beyotime | P0013B | Shanghai | China |
| Reactive Oxygen Species Assay Kit | Beyotime | S0033S | Shanghai | China |
| Recombinant Human IFN γ | Beyotime | P5664 | Shanghai | China |
| Recombinant Human IL-13 | Beyotime | P5178 | Shanghai | China |
| Recombinant Human IL-4 | Beyotime | P5129 | Shanghai | China |
| RPMI-1640 medium | Gibco | 11875093 | New York | USA |
| SYBR-Green Premix Pro Tag HS qPCR Kit | Accurate Biology | AG11702 | Changsha | China |
| TransExo^TM^ Cell Media Exosome Kit | Transgen | FE401-01 | Beijing | China |
| TRIzol™ LS Reagent | Invitgen | 10296010CN | California | USA |
| TRIzol™ Reagent | Invitgen | 15596026 | California | USA |
| β-mercaptoethanol | Sigma-Aldrich | M6250 | Hessen Land | Germany |

Note: The reagents and chemicals arrangement follow from A to Z.

**Table S2. The Information of Antibodies for Protein Simple Wes**

| **Antibody** | **Source** | **Clone** | **Host/** **Subtype** | **Dilution** |
| --- | --- | --- | --- | --- |
| ALIX | Abcam | EPR23653-32 | Rabbit/ IgG | 1:25 |
| CD63 | Abcam | KILL150A | Mouse/ IgG | 1:20 |
| CD80 | NOVUS | OTI2E5 | Mouse/ IgG2a | 1:50 |
| CD86 | NOVUS | BU63 | Mouse/ IgG1 | 1:25 |
| CD163 | Antibodies | Polyclonal | Rabbit/ IgG | 1:25 |
| HSP70 | Abcam | 5A5 | Mouse/ IgG1 | 1:25 |
| IRF5 | Abcam | EPR17067 | Rabbit/ IgG | 1:50 |
| PPARγ | CST | C26H12 | Rabbit/ IgG | 1:50 |
| STAT1 | CST | D1K9Y | Rabbit/ IgG | 1:50 |
| Vinculin | Abcam | EPR8185 | Rabbit/ IgG | 1:500 |
| β-Actin | CST | 13E5 | Rabbit/ IgG | 1:500 |
| β-Actin | CST | 8H10D10 | Mouse/ IgG2b | 1:500 |

Note: The antibody arrangement follows from A to Z.

**Table S3. Oligonucleotides**

| **Name** | **Sequences (5’-3’)** | **Application** |
| --- | --- | --- |
| Has-miR mimic-NC | Sense：UUUGUACUACACAAAAGUACUG | Cell Transfection |
|  | Antisense：CAGUACUUUUGUGUAGUACAAA |  |
| Has-miR-410-5p mimic | Sense：AGGUUGUCUGUGAUGAGUUCG | Cell Transfection |
|  | Antisense：CGAACUCAUCACAGACAACCU |  |
| Has-miR inhibitor-NC | CAGUACUUUUGUGUAGUACAAA | Cell Transfection |
| Has-miR-410-5p inhibitor | UCCAACAGACACUACUCAAGC | Cell Transfection |
| Mmu-agomir-NC | Sense：UUUGUACUACACAAAAGUACUG | Mice Injection Treatment |
|  | Antisense：CAGUACUUUUGUGUAGUACAAA- Chol |  |
| Mmu-agomir-410-5p | Sense：AGGUUGUCUGUGAUGAGUUCG | Mice Injection Treatment |
|  | Antisense：CGAACUCAUCACAGACAACCU-- Chol |  |
| Has-NC-probe | UCACAACCUCCUAGAAAGAGUAGA- Biotin-linker | MiRNA-pulldown |
| Has-miR-410-5p-probe | AGGUUGUCUGUGAUGAGUUCG- Biotin-linker | MiRNA-pulldown |
| Has-siSTAT1-1 | Sense：GUAUAGAGCAUGAAAUCAA (dT)(dT) | Plasmid construction/ Transfection |
|  | Antisense：UUGAUUUCAUGCUCUAUAC (dT)(dT) |  |
| Has-siSTAT1-2 | Sense：GAAGUAUCUGUAUCCAAAU (dT)(dT) | Plasmid construction/ Transfection |
|  | Antisense：AUUUGGAUACAGAUACUUC (dT)(dT) |  |
| Has-siSTAT1-3 | Sense：CGAACAUGACCCUAUCACA (dT)(dT) | Plasmid construction/ Transfection |
|  | Antisense：UGUGAUAGGGUCAUGUUCG (dT)(dT) |  |

Abbreviations: Has, Homo sapiens; Mmu, Mus musculus; NC, negative control.

**Table S4. The Sequences of Primers for Quantitative RT-PCR**

| **Gene Symbol** | **Forward primer (5’-3’)** | **Reverse primer (5’-3’)** |
| --- | --- | --- |
| **Homo sapiens** | | |
| ACTIN | GCCTTTGCCGATCCGC | GCCGTAGCCGTTGTCG |
| CPT1a | TCCAGTTGGCTTATCGTGGTG | TCCAGAGTCCGATTGATTTTTGC |
| CPT2 | CTGGAGCCAGAAGTGTTCCAC | AGGCACAAAGCGTATGAGTCT |
| G6PD | CTGTGCGAGCCGTGCG | CGGGTCTGAGAGTGGG |
| HK2 | GAGTITGACCTGGATGTGGTTGC | CCTCCATGTAGCAGGCATIGCI |
| HK3 | GTGAGGTTGGGCTAGTTGTAGA | GTCCAGGGTATGGTCGAAGGT |
| IL-10 | AACAAGAGCAAGGCCGTGG | GAAGATGTCAAACTCACTCATGGC |
| IL-12 | AAAGGACATCTGCGAGGAAAGTTC | CGAGGTGAGGTGCGTTTATGC |
| IL-23 | TTATGAGAAGCTGCTAGGATCG | GAAGGATTTTGAAGCGGAGAAG |
| LDHA | CACCAGCAACATTCATTCCA | AGCCCGATTCCGTTACCT |
| Mrc1 | CTCTGTTCAGCTATTGGACGC | CGGAATTTCTGGGATTCAGCTTC |
| Rab27a | CAAACAGCTTCCAGCTAAGGAC | GAGAACTCTGTGCCTCACCTCA |
| Rab27b | GACACTGCGGGACAAGAGC | CTTGCCGTTCATTGACTTCC |
| STAT1 | TGTATGCCATCCTCGAGAGC | AGACATCCTGCCACCTTGTG |
| TGF-β | AAGGACCTCGGCTGGAAGTGC | CCGGGTTATGCTGGTTGTA |
| TNF-α | TCTCGAACCCCGAGTGACAA | TGAAGAGGACCTGGGAGTAG |
| **Mus musculus** | | |
| ACTIN | AGGCATCCTCACCCTGAAGTA | CACACGCAGCTCATTGTAGA |
| IL-10 | TGGCCCAGAAATCAAGGAGC | CAGCAGACTCAATACACACT |
| IL-23 | AACAGCTTAAGGATGCCCAGGTTC | ATAATGGTGTCCTTGCCCTTCACG |
| IFN-γ | TGGCATAGATGTGGAAGAAAAGAG | TGCAGGATTTTCATGTCACCAT |
| TGF-β | CACTGATACGCCTGAGTG | GTGAGCGCTGAATCGAAA |
| **miRNA** | | |
| Pre-miR-410 | GTCTGTTGATGAGTTCGCTTTTA | CTCAACTGGTGTCGTGGA |
| miR-410-5p | AGGTTGTCTGTGATGAGTTCG | CTCAACTGGTGTCGTGGA |
| miRNA-RT | CTCAACTGGTGTCGTGGAGTCGGCAATTCAGTTGAGGGTACTGA | |
| U6 | CTCGCTTCGGCAGCACA | AACGCTTCACGAATTTGCGT |

Note: The primer sequences follow from A to Z.

**Table S5. Summary of Reagents for Immunofluorescence**

| **Reagents** | **Source** | **Clone** | **Host/ Subtype** | **Dilution** | **Excitation/Emission Peak (nm)** |
| --- | --- | --- | --- | --- | --- |
| **Primary Antibodies** | | | | | |
| CD68 | Invitrogen | KP1 | Mouse/ IgG1 | 1:100 |  |
| CD80 | NOVUS | OTI2E5 | Rabbit/ IgG2a | 1:100 |  |
| CD163 | Invitrogen | GHI/61 | Rabbit / IgG1 | 1:500 |  |
| **Secondary Antibodies** | | | | | |
| Alexa Fluor 488 | Invitrogen |  | Goat/IgG (H+L) | 1:500 | 495/519 |
| Alexa Fluor 555 | Invitrogen |  | Donkey/IgG (H+L) | 1:500 | 555/565 |
| **Other Reagents** |  |  |  |  |  |
| DAPI | Beyotime |  |  | 1:1000 | 364/454 |

Abbreviations: DAPI, 4',6-diamidino-2-phenylindole.

**Table S6. The Minimum and Maximum Detectable Concentration of Cytokines**

| **Cytokines** | **Minimum Concentration**  **(pg/mL)** | **Maximum Concentration**  **(pg/mL)** |
| --- | --- | --- |
| IL-4 | 1.312 | 8000 |
| IL-6 | 0.81 | 10000 |
| IL-10 | 0.36 | 11000 |
| IL-23 | 2.18 | 10000 |
| IP-10 | 2.03 | 10000 |
| TARC | 1.1 | 10000 |
| TNF-α | 0.195 | 8000 |

Note: The cytokines follow from A to Z.

**Table S7. Summary of Primary Antibodies and Immunohistochemical Technique**

| **Antibody** | **Source** | **Clone** | **Dilution** | **Antigen retrieval** |
| --- | --- | --- | --- | --- |
| CD86 | NOVUS | BU63 | 1:800 | EDTA (pH 8.95-9.05) |
| CD206 | CST | E6T5J | 1:2000 | Citrate (pH 5.80-6.00) |
| STAT1 | CST | D1K9Y | 1:2500 | Citrate (pH 5.80-6.00) |

Abbreviations: EDTA, Ethylenediaminetetraacetic acid.

**Additional file 1 Figure**

**Figure S1. STAT1 is a potential target for miR-410-5p.**

**
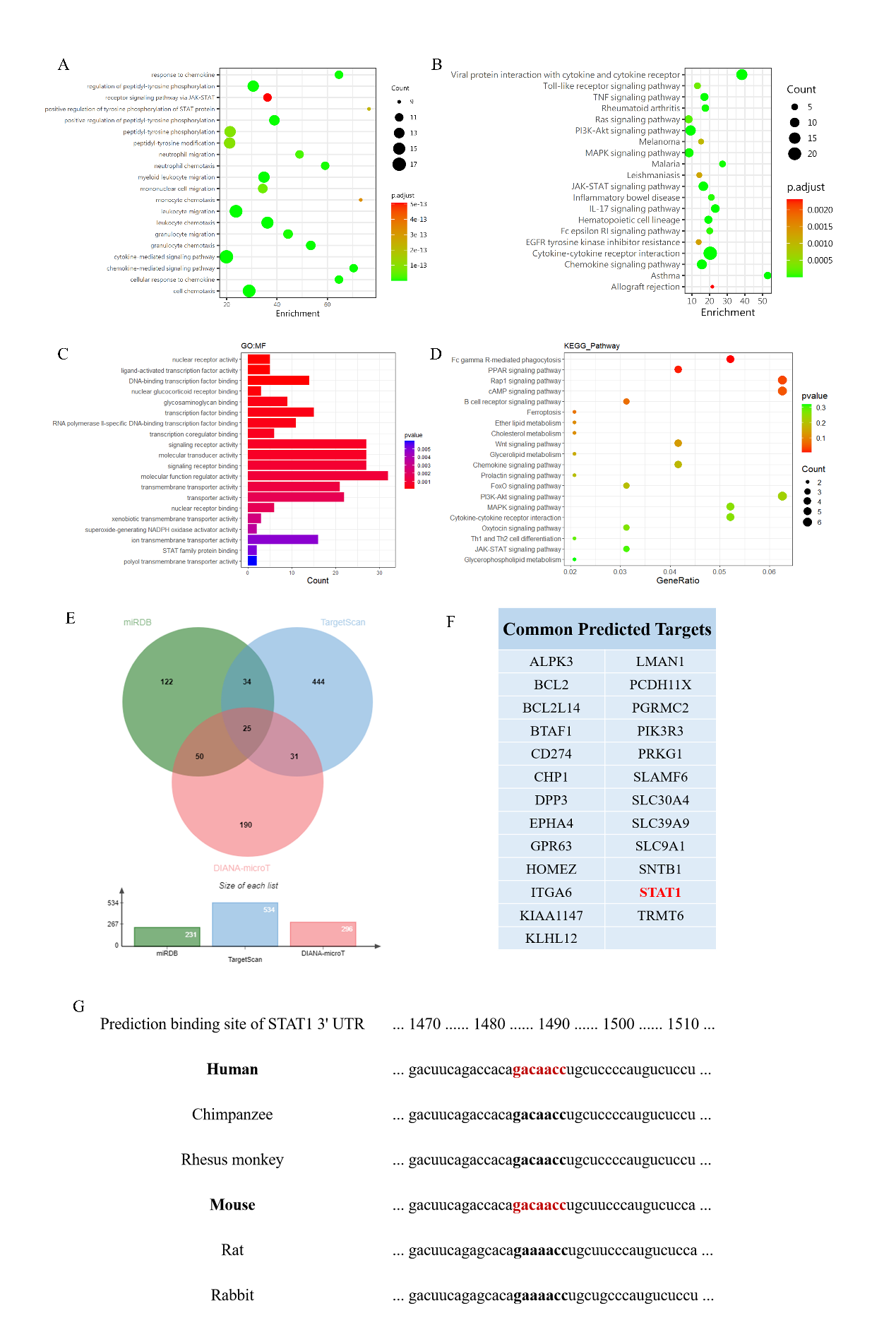
**

**(A-B)** Analysis of the cytokine microarray assay. **(A)** The bubble chart of top 20 GO terms for biological process in M0-miR-410-5p-mimic compared with M0-NC. **(B)** The bubble chart of top 20 KEGG pathways in M0-miR-410-5p-mimic compared with M0-NC. **(C-D)** Analysis of the mRNA sequencing. **(C)** The bar chart of top 20 GO terms for molecular function in M0-miR-410-5p-mimic compared with M0-NC. **(D)** The top 20 KEGG pathways in M0-miR-410-5p-mimic compared with M0-NC. **(E)** Three independent miRNA target databases (miRDB, TargetScan and DIANA-microT) were used to predict miR-410-5p potential mRNA. **(F)** The 25 common target genes in three databases were screened. **(G)** Predicted consequential pairing of miR-410-5p targeting region between different species.
